# Three previously undescribed metabolites from *Cordyceps cicadae* JXCH-1, an entomopathogenic fungus

**DOI:** 10.1007/s13659-023-00410-2

**Published:** 2023-11-03

**Authors:** Jing Fan, Pai Liu, Kuan Zhao, He-Ping Chen

**Affiliations:** 1 School of Pharmaceutical Sciences, South-Central Minzu University, Wuhan, 430074 China; 2 School of Pharmacy, Anhui University of Chinese Medicine, Hefei, 230012 China; 3 College of Life Science, Jiangxi Science & Technology Normal University, Nanchang, 330013 China

**Keywords:** Insect pathogenic fungus, *Cordyceps cicadae*, Steroid, Polyketide, Cyclic dipeptide

## Abstract

Three previously undescribed compounds, cordycicadione (**1**), cordycicadin F (**2**), and 7-hydroxybassiatin (**3**), were isolated from the cultures of *Cordyceps cicadae* JXCH1, an entomopathogenic fungus. Their structures and relative configurations were elucidated primarily by NMR spectroscopic analysis. The absolute configurations of **1** and **2** were determined by ECD calculations. Single-crystal X-ray diffraction method was adopted to determine the absolute configuration of **3**. Compound **2** is a polycyclic polyketide with an unusual enol ether moiety and a spiro ring. The compounds obtained in this study were subjected to screening their inhibition against the proliferation of the human lung cancer cell line A549 and the production of nitric oxide in murine macrophages RAW264.7.

**Graphical Abstract:**

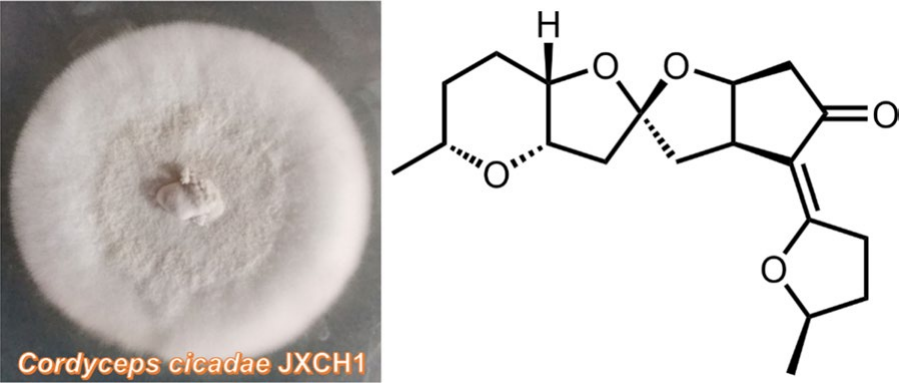

**Supplementary Information:**

The online version contains supplementary material available at 10.1007/s13659-023-00410-2.

## Introduction

The genus *Cordyceps*, a group of insect-pathogenic fungi, includes many species with important medical applications in Chinese Medicine. The sensu stricto species used in Chinese Medicine are *Cordyceps sinensis*, *Cordyceps militaris*, and *Cordyceps cicadae* [1]. Among the three species, *C. sinensis* has been used for treating diverse chronic diseases for more than 300 years, while *C. militaris* is a species which has been widely used as a materia medica, but also used a food supplement. *C. cicadae* is a parasitic fungus formed by cicada nymphs infected by the fungus *C. cicadae* (*= Isaria cicadae*) [2]. *Jin Chan Hua*, a fungus-insect complex used in Chinese Medicine, is one of the earliest medicines recorded in classics of Traditional Chinese Medicine. Pharmacological experiments results suggested that *Jin Chan Hua* is effective remedies for convulsions, chronic kidney diseases, measles, asthma, insomnia, and heart palpitations [3]. However, although the medicinal values of *C. cicadae* have long been recognized, but unlike the *C. militaris*, the *C. cicadae* are not cultivatable [4], and thereby, its shortage of supply has hampered the widely application of this species.

Recently, there have been few studies on the chemical composition and pharmacological effects of the *C. cicadae* strains, which have facilitated the alternative application of the crude fractionated products of this fungus. It is clear that the bioactive substances of *C. cicadae* include nucleosides, polysaccharides, sterols, cyclic dipeptides, amino acids, and other small organic compounds [5–8], which have extensive pharmacological effects including immunomodulatory, antitumor [9], neuroprotective [10], antibacterial [11], and anti-diabetic [12] activities. However, in terms of the exact chemical profiles of the natural products derived from this fungus, only a handful of compounds were reported. Cordycicadins A−D, purified from the fermentation broth of the fungus *C. cicadae* JXCH1, are unprecedented C_20_ polyketides with unusual exocyclic enol ether bridges [3]. Biological evaluations of these polyketides indicate that they showed significant antifeedant activity against silkworm larvae, implying the potential of these compounds in green agrochemicals development. Therefore, the secondary metabolites of *C. cicadae* are indeed worth to be further investigated. Herein, we report the purification as well as structural elucidation of three metabolites from the cultures of the fungus *C. cicadae* JXCH1.

## Results and discussion

**Fig. 1:**
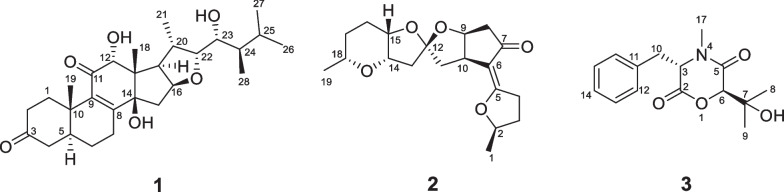
The chemical structures of compounds **1**–**3**

**Fig. 2:**
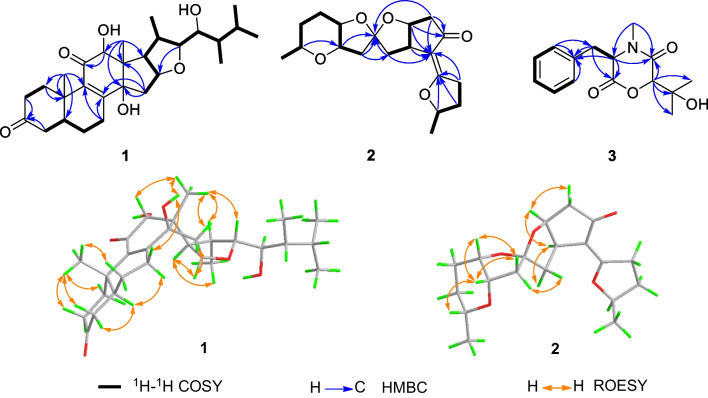
Key 2D NMR correlations of compounds **1**–**3**

**Fig. 3:**
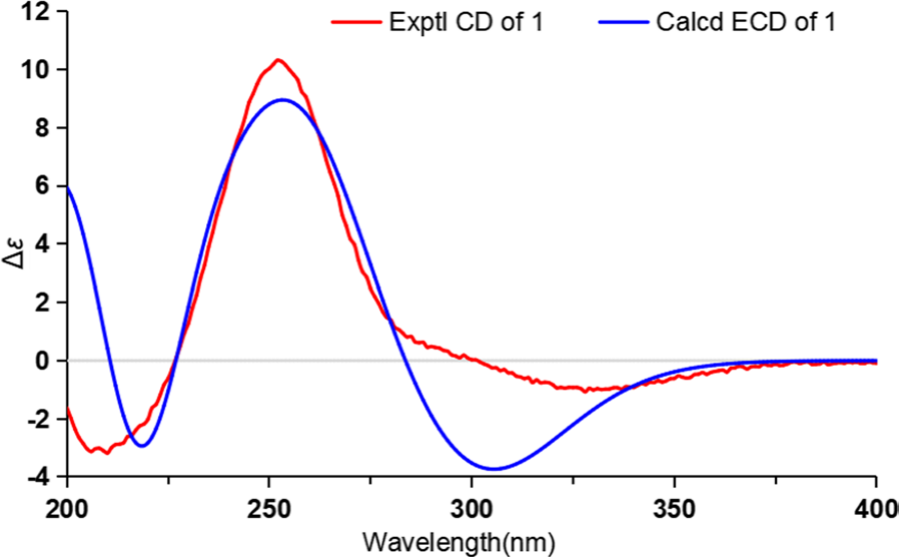
Experimental CD and calculated ECD of **1**

Cordycicadione (**1**, Fig. 1) was obtained as a pale-yellow oil. It presented an ion peak with highest abundance at *m/z* 497.28726 [M + Na]^+^ in the (+)-HRESIMS analysis, which returned a molecular formula of C_28_H_42_O_6_ (calcd for C_28_H_42_O_6_Na, 497.28736), corresponding to eight degrees of unsaturation. The structural elucidation of **1** was accomplished by thorough analysis of the NMR spectra. The ^1^H NMR spectroscopic data of **1** recorded in methanol-*d*_4_ (Table 1) showed resonances for two CH_3_ singlet protons at *δ*_H_ 1.16 (Me-18), 1.28 (Me-19), and four CH_3_ doublets at *δ*_H_ 0.74 (d, *J* = 6.9 Hz, Me-28), 0.76 (d, *J* = 6.9 Hz, Me-26), 0.93 (d, *J* = 7.0 Hz, Me-27), 1.08 (d, *J* = 6.9 Hz, Me-21), four oxygen-attached methine protons at *δ*_H_ 3.30 (overlapped, H-23), 3.76 (s, H-12), 4.19 (br. d, *J* = 7.6 Hz, H-22), 4.49 (m, H-16). The ^13^C NMR as well as DEPT spectroscopic data of **1** recorded in methanol-*d*_4_ (Table 2) displayed sharp carbon signals that were classified into six CH_3_, six CH_2_, nine CH [four oxygenated CH at *δ*_C_ 74.8 (C-23), 80.3 (C-12), 83.5 (C-16) and 85.2 (C-22)], two double bond carbons, three quaternary carbons [sp^3^ hybridized, one oxygenated, *δ*_C_ 83.7 (C-14)], and two ketone carbons at *δ*_C_ 201.0 (C-11) and 214.1 (C-3). The 1D NMR spectroscopic data of **1** displayed similarity with the data of asperfloroid [13], an ergostane steroid purified from the fungus *Aspergillus flocculosus* 16D-1 which resides in sponge, except for the substitutions at C-3, C-5, C-6 and C-7. The C-5 and C-6 are double carbons of the reference compound asperfloroid, while C-5 of **1** is a methine, C-6 and C-7 are methylenes. These changes were substantiated by ^1^H-^1^H COSY spectrum that showed correlations of H_2_-4/H-5/H_2_-6/H_2_-7 (Fig. 2). The HMBC correlations from H_2_-1, H_2_-2, and H_2_-4 to C-3 at *δ*_C_ 214.1 suggested that C-3 was a ketone carbon in **1**, instead of being a hydroxymethine in asperfloroid (Fig. 2). Analysis of the ROESY spectrum recorded in dimethyl sulfoxide-*d*_6_ allowed to determine the relative configuration, which enabled the presence of some pivotal exchangeable protons. The ROESY correlations of H-12/Me-18/OH-14 indicated that OH-14 was β orientation, and OH-12 was α orientation. Moreover, the diagnostic ROESY correlations of Me-18/H-20, Me-18/H-15β (*δ*_H_ 1.82), Me-18/H-22, and H-15α (*δ*_H_ 2.20)/H-16/H-17 suggested the S*and R* configurations of C-16 and C-22, respectively (Fig. 2). The relative stereochemistry of both C-23 and C-24 were assigned as R* by comparing the ^1^H and ^13^C NMR chemicals shifts with those of asperfloroid. Based on above-mentioned analysis, the ECD calculation method was adopted to assign the absolute configuration of **1**. As a result, the calculated ECD of 5* S*,10* S*,12*R*,13* S*,14* S*,16* S*,17*R*,20* S*,22*R*,23*R*,24*R* showed compatible curve with that of the experimental CD spectrum (Fig. 3). Therefore, the evidence presents in above texts help to establish the structure of compound **1** (Fig. 1).

**Table 1:** ^1^H NMR Spectroscopic data of compounds **1**−**3** (600 MHz)

No.	**1**^a^	**1**^b^	**2**^a^	**3**^c^
1	1.49, ddd (13.5, 13.5, 5.5) 3.05, ddd (13.6, 7.2, 2.2)	1.28, ddd (13.5, 13.5, 5.2) 2.81, m	1.40, d (6.2)	
2	2.29, overlapped 2.54, overlapped	2.15, overlapped 2.47, ddd (13.5, 13.5, 7.2)	4.68, m	
3			1.70, m 2.28, m	4.67, t (4.8)
4	2.19, br. d (15.5) 2.48, dd (14.9, 14.8)	2.05, overlapped 2.38, dd (14.5, 14.5)	2.94, ddd (18.5, 8.9, 8.9) 3.30, overlapped	
5	1.81, m	1.66, m		
6	1.53, m 1.56, m	1.39, overlapped 1.41, overlapped		3.10, s
7	2.34, overlapped 2.75, dd (19.6, 5.1)	2.15, overlapped 2.66, dd (19.6, 5.0)		
8			2.36, d (18.5) 2.59, overlapped	1.07, s
9			4.62, overlapped	1.02, s
10			3.61, m	3.28, dd (14.2, 4.8) 3.37, dd (14.2, 4.8)
11			2.10, dd (13.7, 5.1) 2.59, overlapped	
12	3.76, s	3.53, br. s		7.21, overlapped
13			2.04, d (14.5) 2.31, dd (14.5, 4.9)	7.36, overlapped
14			3.99, dd (4.9, 2.1)	7.36, overlapped
15	1.94, dd (13.2, 6.6) 2.39, dd (13.2, 7.2)	1.82, dd (13.4, 5.0) 2.20, dd (13.4, 7.2)	3.89, m	7.36, overlapped
16	4.49, m	4.47, m	1.80, m 2.02, br. d (14.6)	7.21, overlapped
17	2.29, overlapped	2.22, br. t (7.2, 7.2)	1.44, overlapped	3.05, s
18	1.16, s	0.98, s	3.41, m	
19	1.28, s	1.20, s	1.14, d (6.2)	
20	2.56, overlapped	2.49, overlapped		
21	1.08, d (6.9)	1.00, d (6.9)		
22	4.19, br. d (7.6)	3.97, d (7.8)		
23	3.30, overlapped	3.10, t (8.6)		
24	1.64, m	1.52, m		
25	2.11, m	2.07, overlapped		
26	0.76, d (6.9)	0.67, d (6.8)		
27	0.93, d (7.0)	0.84, d (7.0)		
28	0.74, d (6.9)	0.63, d (6.9)		
7-OH				4.80, s
12-OH		5.31, s		
14-OH		4.95, s		
23-OH		4.03, d (7.6)		

^a^Measured in CD_3_OD; ^b^Measured in DMSO-*d*_6_; ^c^Measured in CD_3_COCD_3_

**Table 2:** ^13^C NMR spectroscopic data of compounds **1**−**3** (150 MHz)

No.	**1**^a^	**1**^b^	**2**^a^	**3**^c^
1	36.0, CH_2_	34.3, CH_2_	20.6, CH_3_	
2	38.8, CH_2_	37.5, CH_2_	83.0, CH	166.9, C
3	214.1, C	210.1, C	31.6, CH_2_	63.1, CH
4	44.7, CH_2_	43.5, CH_2_	31.8, CH_2_	
5	45.0, CH	43.0, CH	174.1, C	167.5, C
6	25.4, CH_2_	23.9, CH_2_	111.4, C	80.4, CH
7	27.7, CH_2_	25.9, CH_2_	206.6, C	72.2, C
8	158.8, C	157.3, C	46.3, CH_2_	24.0, CH_3_
9	137.8, C	134.8, C	78.0, CH	27.0, CH_3_
10	37.6, C	35.5, C	44.6, CH	37.4, CH_2_
11	201.0, C	199.6, C	45.6, CH_2_	136.1, C
12	80.3, CH	79.5, CH	117.1, C	130.7, CH
13	51.8, C	51.0, C	45.8, CH_2_	129.8, CH
14	83.7, C	82.1, C	78.3, CH	128.7, CH
15	45.4, CH_2_	45.6, CH_2_	75.8, CH	129.8, CH
16	83.5, CH	82.8, CH	26.1, CH_2_	130.7, CH
17	57.6, CH	56.8, CH	28.3, CH_2_	32.3, CH_3_
18	17.2, CH_3_	16.2, CH_3_	73.1, CH	
19	16.4, CH_3_	16.2, CH_3_	22.2, CH_3_	
20	39.5, CH	38.1, CH		
21	14.9, CH_3_	14.5, CH_3_		
22	85.2, CH	83.0, CH		
23	74.8, CH	72.7, CH		
24	41.7, CH	40.1, CH		
25	27.2, CH	25.4, CH		
26	15.6, CH_3_	15.4, CH_3_		
27	22.2, CH_3_	21.7, CH_3_		
28	10.4, CH_3_	9.9, CH_3_		

^a^Measured in CD_3_OD; ^b^Measured in DMSO-*d*_6_; ^c^Measured in CD_3_COCD_3_

**Fig. 4:**
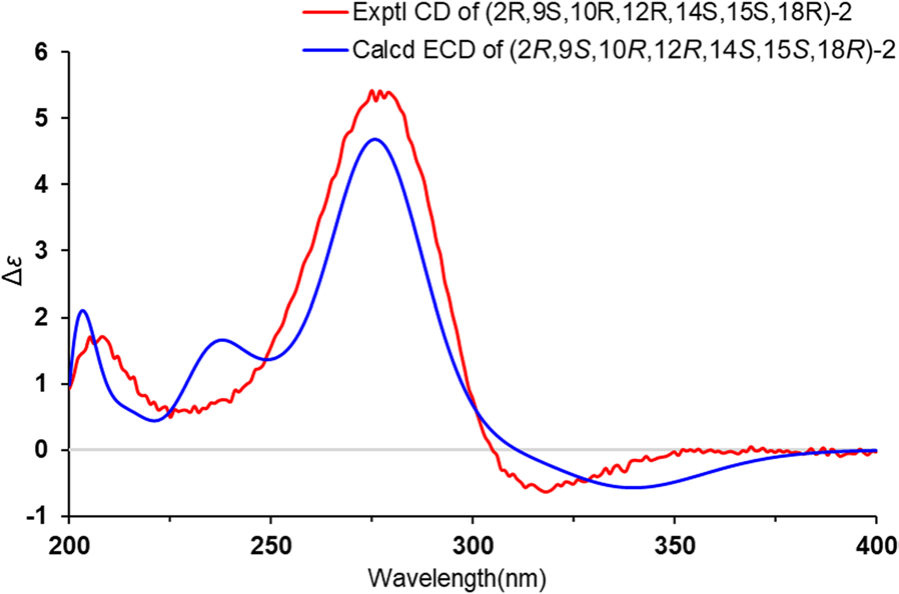
Experimental CD and calculated ECD of **2**

Cordycicadin F (**2**, Fig. 1), a pale-yellow oil, possesses the molecular formula of C_19_H_26_O_5_ from the ion peak at *m/z* 335.18518 [M + H]^+^ (calcd for C_19_H_27_O_5_, 335.18530) in (+)-HRESIMS analysis. The ^1^H NMR spectroscopic data of **2** (Table 1) showed two doublet methyl signals at *δ*_H_ 1.14 (d, *J* = 6.2 Hz, Me-19), 1.40 (d, *J* = 6.2 Hz, Me-1), five oxygen-connected methine protons at *δ*_H_ 3.41 (m, H-18), 3.89 (m, H-15), 3.99 (dd, *J* = 4.9, 2.1 Hz, H-14), 4.62 (overlapped, H-9), 4.68 (m, H-2). The ^13^C NMR along with the DEPT spectroscopic data of **2** (Table 2) indicated resonances for 19 carbons attributable to two CH_3_ carbon at *δ*_C_ 20.6 (C-1) and 22.2 (C-19), seven CH_2_ carbons at *δ*_C_ 26.1 (C-16), 28.3 (C-17), 31.6 (C-3), 31.8 (C-4), 45.6 (C-11), 45.8 (C-13), 46.3 (C-8), six methines [five oxygenated ones at *δ*_C_ 73.1 (C-18), 75.8 (C-15), 78.0 (C-9), 78.3 (C-14), and 83.0 (C-2)], one pair of double bonds at *δ*_C_ 111.4 (C-6) and 174.1 (C-5), one ketal carbon and one ketone carbon at *δ*_C_ 117.1 (C-12) and *δ*_C_ 206.6 (C-7), respectively. These data of **2** (Tables 1 and 2) resembled those of the known compound opaliferin [14], a polyketide isolated from the entomopathogenic fungus *Cordyceps* sp. NBRC 106954. The difference between compound **2** and opaliferin is that the C-14 and C-18 of opaliferin are each substituted with a hydroxy group, while the C-14 and C-18 of **2** are attached by an ether bond. This change was reinforced by the HMBC correlations from H-18 to C-14 (Fig. 2). The tetrahydrofuran moiety (C-1–C-5) was established by HMBC correlations from H-2, H_2_-3, and H_2_-4 to C-5. The presence of cyclopentanone moiety (C-6–C-10) was confirmed by the HMBC correlations of H-8, H-9 to C-6, C-7, and of H-10, H-11 to C-6. The HMBC correlation from H_2_-4 to C-6 and chemical shifts of C-5 (*δ*_C_ 174.1), C-6 (*δ*_C_ 111.4) in ^13^C NMR spectrum suggested the existence of an enol moiety. The HMBC correlations from H_2_-11 to C-12, H_2_-13 to C-11, H_2_-13 to C-12, H-14 to C-12, H-15 and H-9 to C-12, together with the chemical shift of C-12 (*δ*_C_ 117.1) indicated that C-12 was an acetal carbon which connected to C-9 and C-15 by two epoxy bonds. All of these data were highly similar to opaliferin. Furthermore, the configurations of the chiral carbons of **2** were consistent with those of opaliferin, which were assigned as 2*R*,5*E*,9*S*,10*R*,12*R*,14*S*,15*S*,18*R* by biosynthetic considerations, comparison with the NMR data, and the ROESY correlations of H-14/H-15/H-18, and H-9/H-10 (Fig. 2), and confirmed by ECD calculation (Fig. 4). Therefore, compound **2** was established and was trivially named cordycicadin F.

**Fig. 5:**
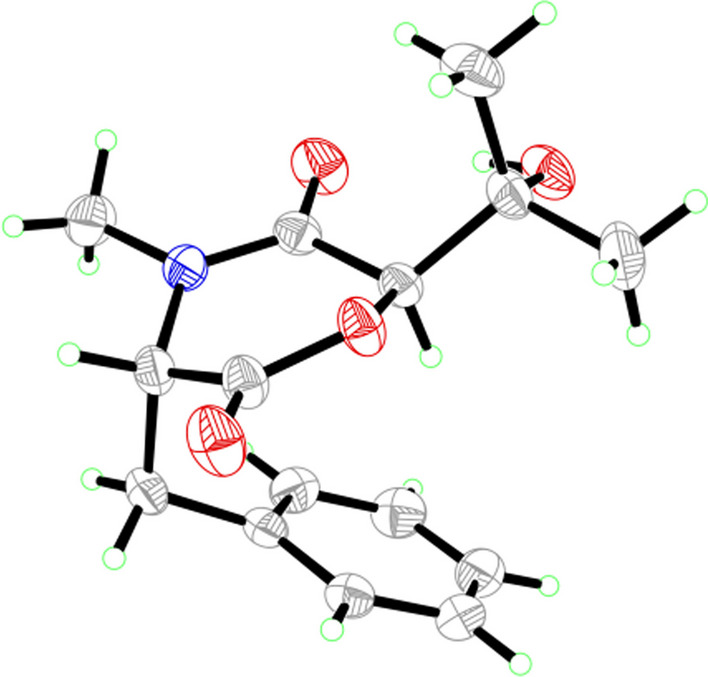
X-ray ORTEP drawing of compound **3**

The molecular formula of the colorless crystals 7-hydroxybassiatin (**3**, Fig. 1) was determined as C_15_H_19_NO_4_ by the protonated ion peak at *m/z* 278.13851 [M + H]^+^ (calcd for C_15_H_20_NO_4_, 278.13868) in the HRESIMS analysis, requiring seven degrees of double bond equivalence. The proton NMR data of **3** (Table 1) showed three singlet methyls [two methyl protons at *δ*_H_ 1.02 (Me-9) and 1.07 (Me-8), one *N*-methyl protons at *δ*_H_ 3.05 (Me-17)], two methine protons at *δ*_H_ 3.10 (s, H-6) and 4.67 (t, *J* = 4.8 Hz, H-3), one pair of methylene protons at *δ*_H_ 3.28 (dd, *J* = 14.2, 4.8 Hz, H-10), 3.37 (dd, *J* = 14.2, 4.8 Hz, H-10), one exchangeable proton at *δ*_H_ 4.80 (s, OH-7), and five aromatic protons. The ^13^C NMR and DEPT spectroscopic data of **2** (Table 2) presented carbon signals for three methyl carbons at *δ*_C_ 24.0 (C-8), 27.0 (C-9) and 32.3 (C-17), one methylene carbon at *δ*_C_ 37.4 (C-10), seven methines [one oxygenated at *δ*_C_ 80.4 (C-6), the others at *δ*_C_ 63.1 (C-3), 128.7 (C-14), 129.8 (C-13), 129.8 (C-15), 130.7 (C-12), 130.7 (C-16)], two quaternary carbons at *δ*_C_ 72.2 (C-7) and 136.1 (C-11), and two ketone carbons at *δ*_C_ 166.9 (C-2) and 167.5 (C-5). The aforementioned data exhibited high similarity to bassiatin, which was isolated from the cultured broth of the fungus *Beauveria bassiana* K-717 [15]. The 2D NMR spectroscopic analysis revealed that the major structural discrepancy between **3** and bassiatin was that the substituted pattern of C-7. The C-7 of bassiatin is a methine while C-7 of **3** is attached by a hydroxy group. The difference was fully corroborated by the HMBC correlations from OH-7 to C-6, C-7 and C-9. Therefore, the absolute configuration of **3** was assigned as 3*S*,6*R* by single-crystal X-ray diffraction analysis with the Flack parameter of 0.01(4) (Fig. 5). Hence, the structure of compound **3** was established (Fig. 1), and trivially named 7-hydroxybassiatin.

The compounds isolated were tested for their cytotoxicity toward A549 cell line (human lung cancer), and the anti-inflammatory activity in murine RAW264.7 macrophages by evaluating the production of nitric oxide (NO). However, the results suggested that they were devoid of any activity.

## Conclusion

Three new compounds isolated from the cultures of the entomopathogenic fungus *C. cicadae* JXCH1. The isolates in this study were subjected to screening their inhibition against the proliferation of the A549, a human lung cancer cell line, and the production of nitric oxide in murine macrophages RAW264.7. Although they were devoid of any activity in the biological activity screening model used in this study, but these compounds may play important or untapped roles during the process of fungal invasion into the insect hosts. Besides, this study extends the diversity of secondary metabolites in the fungus *C. cicadae*, which also promotes the understanding of chemical basis of traditional Chinese medicine. In subsequent experiments, we also hope to find molecules with novel skeleton or better activity from *C. cicadae* to provide more lead compounds for drug research and development.

## Experimental section

### General experimental procedures

The laboratory instruments used in this study are as same as those reported in the literature [16–18].

### Fungal material

A flower-like cicadae larva-fungi complex was collected in Dajueshan Natural Reserve, Fuzhou, Jiangxi Province, China, in June 2018. The “flower” part of the complex was wash by disinfectant distilled water for three times. The inner part of the “flower” which contained powder-like spores was transferred onto the potato-dextrose-agar (PDA) plate supplemented with ampicillin and streptomycin. The fungus was purified by several times of efforts, and it was identified by sequencing the internal transcribed spacer region, which was amplified from the genomic DNA of this fungus with the primers ITS1 and ITS4. As a result, the sequencing data (GenBank Accession No. OP591391) suggest that the fungus was *C. cicadae*. A voucher specimen of the cicadae larva-fungi complex and the fungus (JXCH1) was kept by the Mushroom Bioactive Natural Products Research Group of South-Central Minzu University.

The fresh strain of *C. cicadae* JXCH1 was grew on PDA medium for seven days (25 ℃). Then the plate agar was cut into small cubes to inoculate 200 Erlenmeyer flasks (500 mL), each containing 250 mL of culture medium which consists of 5 g glucose, 1.5 g pork peptone, 5 g yeast extract, 0.5 g KH_2_PO_4_, 0.5 g MgSO_4_ per liter. The flasks were cultured on dark shakers at 25 °C and 160 rpm for 25 days.

### Extraction and isolation

Based on previous experience on handling the cultures of this fungus [3], 50 L of methanol was added into the cultures and soaked for more than 3 days in room temperature, followed by centrifugation to break apart the mycelia and broth. The liquid phase was evaporated in vacuo to 3 L, and it was further partitioned with ethyl acetate (EtOAc) (3 × 3 L). The EtOAc layer was gathered and concentrated to give the extract A (20.2 g). The mycelia part was soaked in 5 L MeOH (3 × 3 days). The MeOH extract was combined and removed in reduced pressure, then dissolved in water and partitioned against EtOAc to give the extract B (33.3 g). The total crude extract (A + B, 53.5 g) was fractionated by MPLC with a stepwise gradient of MeOH-H_2_O (20:80–100:0) to afford fifteen fractions (A–O).

Fraction I was subjected to a size-exclusion CC (Sephadex LH-20) eluting with acetone and yielded ten subfractions (I1–I10). Subfraction I2 further separated by prep-HPLC (H_2_O-MeCN: 75:25–50:50, 25 min, 4 mL min^−1^) to gain six subfractions (I2a–I2f). Subfraction I2b was separated into three subfractions (I2b1–I2b3) by prep-HPLC (H_2_O-MeOH: 60:40–40:60, 30 min, 3 mL min^−1^). Subfraction I2b1 was purified on prep-HPLC (H_2_O-MeCN: 69:31–57:43, 15 min, 4 mL min^−1^) to yield compounds **3** (t_*R*_ = 14.5 min, 3.9 mg).

Fractions K-M were combined and subjected to a size-exclusion CC (Sephadex LH-20) eluting with acetone and yielded fifteen subfractions (K1–K15). Compounds **1** (t_*R*_ = 23.26 min, 2.4 mg) and **2** (t_*R*_ = 19.55 min, 3.0 mg) were isolated from subfraction K6 by prep-HPLC (H_2_O-MeCN: 70:30–46:54, 30 min, 4 mL min^−1^).

### Characterization data

#### Cordycicadione (**1**)

Pale-yellow oil; ${{\left[\alpha \right]}^{23.0}_{\text{D}}}$  + 46.9 (*c* 0.05, MeOH); UV (MeOH) λ_max_ (log *ε*) 245.0 (3.50) nm; ^1^H NMR (600 MHz, CD_3_OD and DMSO-*d*_6_) data, see Table 1, ^13^C NMR (150 MHz, CD_3_OD and DMSO-*d*_6_) data, see Table 2; HRESIMS *m/z* 497.28726 [M + Na]^+^ (calcd for C_28_H_42_O_6_Na, 497.28736).

#### Cordycicadin F (**2**)

Pale-yellow oil; ${{\left[\alpha \right]}^{25.0}_{\text{D}}}$ + 17.3 (*c* 0.05, MeOH); UV (MeOH) λ_max_ (log *ε*) 280.0 (3.97) nm; ^1^H NMR (600 MHz, CD_3_OD) data, see Table 1, ^13^C NMR (150 MHz, CD_3_OD) data, see Table 2; HRESIMS *m/z* 335.18518 [M + H]^+^ (calcd for C_19_H_27_O_5_, 335.18530).

#### 7-Hydroxybassiatin (**3**)

Colorless crystals; ${{\left[\alpha \right]}^{25.0}_{\text{D}}}$ + 14.2 (*c* 0.05, MeOH); UV (MeOH) λ_max_ (log *ε*) 210.0 (4.03) nm; ^1^H NMR (600 MHz, CD_3_COCD_3_) data, see Table 1, ^13^C NMR (150 MHz, CD_3_COCD_3_) data, see Table 2; HRESIMS *m/z* 278.13851 [M + H]^+^ (calcd for C_15_H_20_NO_4_, 278.13868).

### Computational details

The Gaussian 16 package was used as the software for all the calculation jobs [19]. Firstly, conformational analyses of the candidate molecules were searched at MMFF94s force field. The returned conformers which occupy more than 1% proportion were optimized by the density functional theory (DFT) method at the B3LYP/6-31G(d) level. The optimized conformers were subjected to electronic CD calculations. The method used in ECD calculation was time-dependent DFT (TD-DFT) at B3LYP/6-31G(d,p) level, the solvent model was IEFPCM model in MeOH (Additional file 1). The ECD calculation results were processed by the SpecDis software (v1.71) [20], the calculated ECD and experimental CD curves were plotted in the Microsoft Office Excel program.

### Single-crystal X-ray diffraction data of 3

A colorless block-like sample with the molecular formula of C_15_H_19_NO_4_, *M* = 277.31, approximate dimensions 0.228 mm × 0.247 mm × 0.322 mm, was used for the X-ray crystallographic analysis on the BRUKER D8 QUEST diffractometer. The integration of the data using an orthorhombic unit cell yielded a total of 20,646 reflections to a maximum *θ* angle of 79.51° (0.78 Å resolution), of which 3218 were independent (average redundancy 6.416, completeness = 99.4%, *R*_int_ = 3.45%, *R*_sig_ = 2.93%) and 3160 (98.20%) were greater than 2σ(*F*^2^). The final cell constants of *a* = 6.7753(4) Å, *b* = 12.8266(8) Å, *c* = 17.1279(11) Å, *α* = 90.00°, *β* = 90.00°, *γ* = 90.00°, volume = 1488.48(16) Å^3^, T = 273(2) K. Data were corrected for absorption effects using the Multi-Scan method (SADABS). The structure was solved and refined using the Bruker SHELXTL Software Package, using the space group *P2*_1_*2*_1_*2*_1_, with Z = 4, µ(Cu*K*α) = 1.54178. The final anisotropic full-matrix least-squares refinement on *F*^2^ with 189 variables converged at R1 = 2.87%, for the observed data and *wR*^2^ = 7.66% for all data. The goodness-of-fit was 1.053. The absolute configuration was determined by the Flack parameter = 0.01(4), which was determined using 1303 quotients [(I+)-(I−)]/[(I+)+(I−)] [21]. The crystallographic data have been deposited to the Cambridge Crystallographic Data Centre (CCDC 2250285).

### Biological activity assays

#### Anti-proliferative assay

The anti-proliferative of the isolated compounds in this study against the five cancer cell lines were conducted by the same protocol in Ref. [22].

#### Anti-inflammatory activity assay

The anti-inflammatory of the isolated compounds against the murine RAW264.7 macrophages were conducted by the same protocol in Ref. [22].

### Supplementary Information

**Additional file 1: Figure S1.** ^1^H NMR spectrum of compound **1** (600 MHz, CD_3_OD). **Figure S2**. ^13^C and DEPT NMR spectra of compound **1** (150 MHz, CD_3_OD). **Figure S3**. HSQC spectrum of compound **1** (CD_3_OD). **Figure S4**. ^1^H-^1^H COSY spectrum of compound **1** (CD_3_OD). **Figure S5**. HMBC spectrum of **1** (CD_3_OD). **Figure S6**. HRESIMS report of compound **1**. **Figure S7**. ^1^H NMR spectrum of compound **1** (600 MHz, DMSO-*d*_6_). **Figure S8**. ^13^C and DEPT NMR spectra of compound **1** (150 MHz, DMSO-*d*_6_). **Figure S9**. HSQC spectrum of compound **1** (DMSO-*d*_6_). **Figure S10**. ^1^H-^1^H COSY spectrum of compound **1** (DMSO-*d*_6_). **Figure S11**. HMBC spectrum of compound **1** (DMSO-*d*_6_). **Figure S12**. ROESY spectrum of compound **1** (DMSO-*d*_6_). **Figure S13**. ^1^H NMR spectrum of compound **2** (600 MHz, CD_3_OD). **Figure S14**. ^13^C and DEPT NMR spectra of compound **2** (150 MHz, CD_3_OD). **Figure S15**. HSQC spectrum of compound **2** (CD_3_OD). **Figure S16**. ^1^H-^1^H COSY spectrum of compound **2** (CD_3_OD). **Figure S17**. HMBC spectrum of compound **2** (CD_3_OD). **Figure S18**. ROESY spectrum of compound **2** (CD_3_OD). **Figure S19**. HRESIMS report of compound **2**. **Figure S20**. ^1^H NMR spectrum of compound **3** (600 MHz, CD_3_COCD_3_). **Figure S21**. ^13^C and DEPT NMR spectra of compound **3** (150 MHz, CD_3_COCD_3_). **Figure S22**. HSQC spectrum of compound **3** (CD_3_COCD_3_). **Figure S23**. ^1^H-^1^H COSY spectrum of compound **3** (CD_3_COCD_3_). **Figure S24**. HMBC spectrum of compound **3** (CD_3_COCD_3_). **Figure S25**. ROESY spectrum of compound **3** (CD_3_COCD_3_). **Figure S26**. HRESIMS report of compound **3**. **Figure S27**. CD spectrum of **1**. **Figure S28**. CD spectrum of compound **2**. **Figure S29**. CD spectrum of compound **3**. ECD calculation details of compounds **1** and **2**.

## Data Availability

All data generated and analyzed during this study are included in this published article and its Additional file 1.
