# Supplementary material for: Three previously undescribed metabolites from Cordyceps cicadae JXCH-1, an entomopathogenic fungus
Source: Nat Prod Bioprospect. 2023 Nov 3;13(1):46. doi: 10.1007/s13659-023-00410-2 (PMC10622384; doi:10.1007/s13659-023-00410-2)
Supplement: Supplementary file 1 — Additional file 1: Figure S1. 1H NMR spectrum of compound 1 (600 MHz, CD3OD). Figure S2. 13C and DEPT NMR spectra of compound 1 (150 MHz, CD3OD). Figure S3. HSQC spectrum of compound 1 (CD3OD). Figure S4. 1H-1H COSY spectrum of compound 1 (CD3OD). Figure S5. HMBC spectrum of 1 (CD3OD). Figure S6. HRESIMS report of compound 1. Figure S7. 1H NMR spectrum of compound 1 (600 MHz, DMSO-d6). Figure S8. 13C and DEPT NMR spectra of compound 1 (150 MHz, DMSO-d6). Figure S9. HSQC spectrum of compound 1 (DMSO-d6). Figure S10. 1H-1H COSY spectrum of compound 1 (DMSO-d6). Figure S11. HMBC spectrum of compound 1 (DMSO-d6). Figure S12. ROESY spectrum of compound 1 (DMSO-d6). Figure S13. 1H NMR spectrum of compound 2 (600 MHz, CD3OD). Figure S14. 13C and DEPT NMR spectra of compound 2 (150 MHz, CD3OD). Figure S15. HSQC spectrum of compound 2 (CD3OD). Figure S16. 1H-1H COSY spectrum of compound 2 (CD3OD). Figure S17. HMBC spectrum of compound 2 (CD3OD). Figure S18. ROESY spectrum of compound 2 (CD3OD). Figure S19. HRESIMS report of compound 2. Figure S20. 1H NMR spectrum of compound 3 (600 MHz, CD3COCD3). Figure S21. 13C and DEPT NMR spectra of compound 3 (150 MHz, CD3COCD3). Figure S22. HSQC spectrum of compound 3 (CD3COCD3). Figure S23. 1H-1H COSY spectrum of compound 3 (CD3COCD3). Figure S24. HMBC spectrum of compound 3 (CD3COCD3). Figure S25. ROESY spectrum of compound 3 (CD3COCD3). Figure S26. HRESIMS report of compound 3. Figure S27. CD spectrum of 1. Figure S28. CD spectrum of compound 2. Figure S29. CD spectrum of compound 3. ECD calculation details of compounds 1 and 2. [file 13659_2023_410_MOESM1_ESM.docx]

**Additional file**

**Three Previously Undescribed Metabolites from the Insect Pathogenic Fungus *Cordyceps cicadae* JXCH-1**

Jing Fan^1,4^, Pai Liu^2,4^, Kuan Zhao^3,^*, He-Ping Chen^1,^*

^1^School of Pharmaceutical Sciences, South-Central Minzu University, Wuhan 430074, China

^2^School of Pharmacy, Anhui University of Chinese Medicine, Hefei 230012, China

^3^College of Life Science, Jiangxi Science & Technology Normal University, Nanchang 330013, China

^4^These authors contributed equally to this work.

Emails: 1020160918@jxstnu.edu.cn (K. Zhao); [chenhp@mail.scuec.edu.cn](mailto:chenhp@mail.scuec.edu.cn) (H.-P. Chen)

**Content**

[1. Supplementary figures of compounds **1**–**3**. 3](#_Toc16331)

[Figure S1. ^1^H NMR spectrum of **1** (600 MHz, CD_3_OD). 3](#_Toc17669)

[Figure S2. ^13^C and DEPT NMR spectra of **1** (150 MHz, CD_3_OD). 3](#_Toc7067)

[Figure S3. HSQC spectrum of **1** (CD_3_OD). 4](#_Toc11259)

[Figure S4. ^1^H-^1^H COSY spectrum of **1** (CD_3_OD). 4](#_Toc10663)

[Figure S5. HMBC spectrum of **1** (CD_3_OD). 5](#_Toc8876)

[Figure S6. HRESIMS report of **1**. 6](#_Toc9777)

[Figure S7. ^1^H NMR spectrum of **1** (600 MHz, DMSO-*d*_6_). 7](#_Toc16853)

[Figure S8. ^13^C and DEPT NMR spectra of **1** (150 MHz, DMSO-*d*_6_). 7](#_Toc26235)

[Figure S9. HSQC spectrum of **1** (DMSO-*d*_6_). 8](#_Toc4934)

[Figure S10. ^1^H-^1^H COSY spectrum of **1** (DMSO-*d*_6_). 8](#_Toc30818)

[Figure S11. HMBC spectrum of **1** (DMSO-*d*_6_). 9](#_Toc4994)

[Figure S12. ROESY spectrum of **1** (DMSO-*d*_6_). 9](#_Toc31732)

[Figure S13. ^1^H NMR spectrum of **2** (600 MHz, CD_3_OD). 10](#_Toc2456)

[Figure S14. ^13^C and DEPT NMR spectra of **2** (150 MHz, CD_3_OD). 10](#_Toc28274)

[Figure S15. HSQC spectrum of **2** (CD_3_OD). 11](#_Toc11909)

[Figure S16. ^1^H-^1^H COSY spectrum of **2** (CD_3_OD). 11](#_Toc6810)

[Figure S17. HMBC spectrum of **2** (CD_3_OD). 12](#_Toc10461)

[Figure S18. ROESY spectrum of **2** (CD_3_OD). 12](#_Toc16446)

[Figure S19. HRESIMS report of **2**. 13](#_Toc18065)

[Figure S20. ^1^H NMR spectrum of **3** (600 MHz, CD_3_COCD_3_). 14](#_Toc21857)

[Figure S21. ^13^C and DEPT NMR spectra of **3** (150 MHz, CD_3_COCD_3_). 14](#_Toc28170)

[Figure S22. HSQC spectrum of **3** (CD_3_COCD_3_). 15](#_Toc5493)

[Figure S23. ^1^H-^1^H COSY spectrum of **3** (CD_3_COCD_3_). 15](#_Toc18672)

[Figure S24. HMBC spectrum of **3** (CD_3_COCD_3_). 16](#_Toc30586)

[Figure S25. ROESY spectrum of **3** (CD_3_COCD_3_). 16](#_Toc32342)

[Figure S26 HRESIMS report of **3**. 17](#_Toc10756)

[Figure S27. CD spectrum of **1**. 18](#_Toc16166)

[Figure S28. CD spectrum of **2**. 18](#_Toc27759)

[Figure S29. CD spectrum of **3**. 18](#_Toc16122)

[2. Calculation details. 19](#_Toc17617)

[2.1 ECD calculation of **1**. 19](#_Toc28271)

[2.2 ECD calculation of **2**. 22](#_Toc6709)

# Supplementary figures of compounds 1–3.

## Figure S1. ^1^H NMR spectrum of 1 (600 MHz, CD_3_OD).


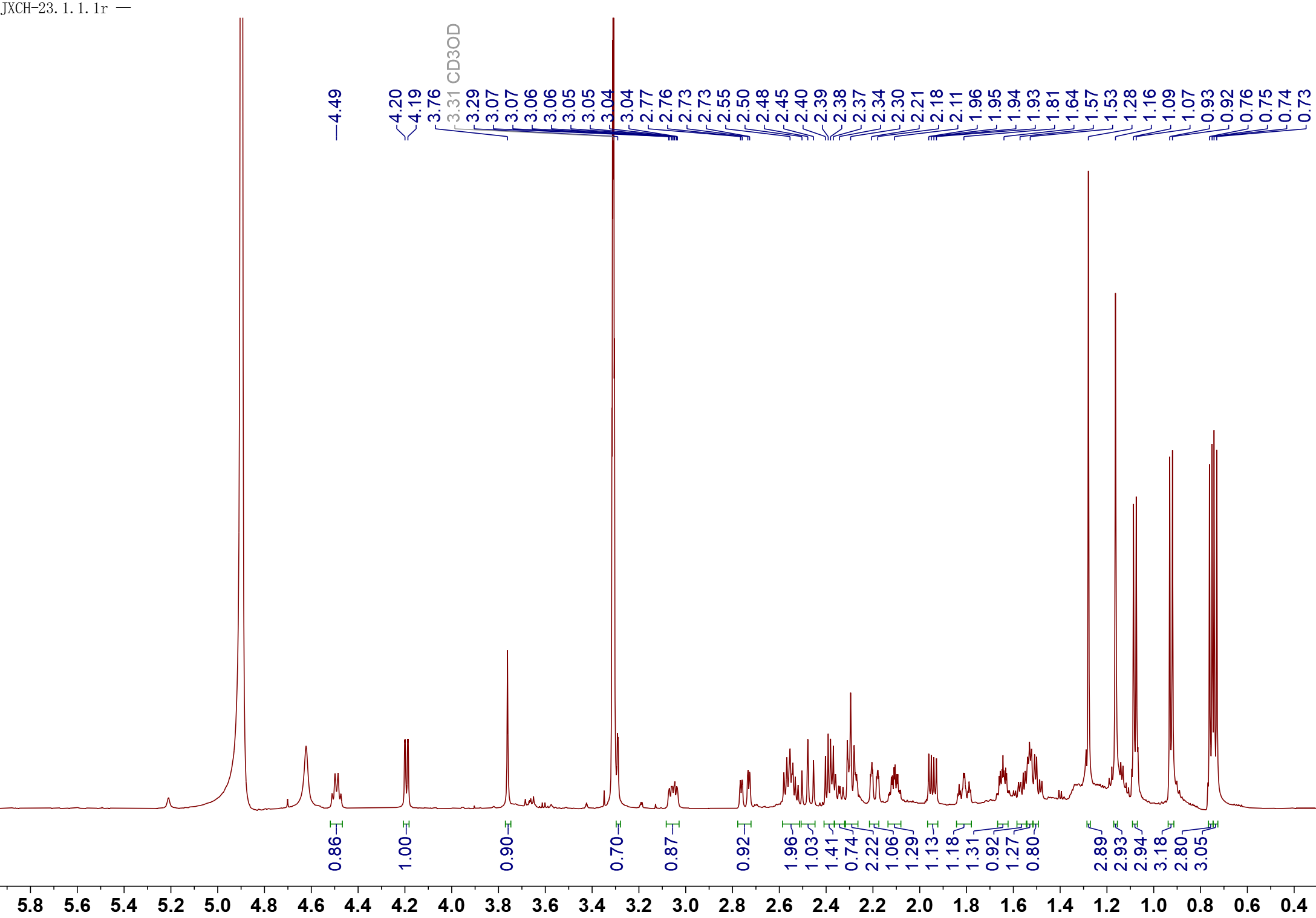


## Figure S2. ^13^C and DEPT NMR spectra of 1 (150 MHz, CD_3_OD).


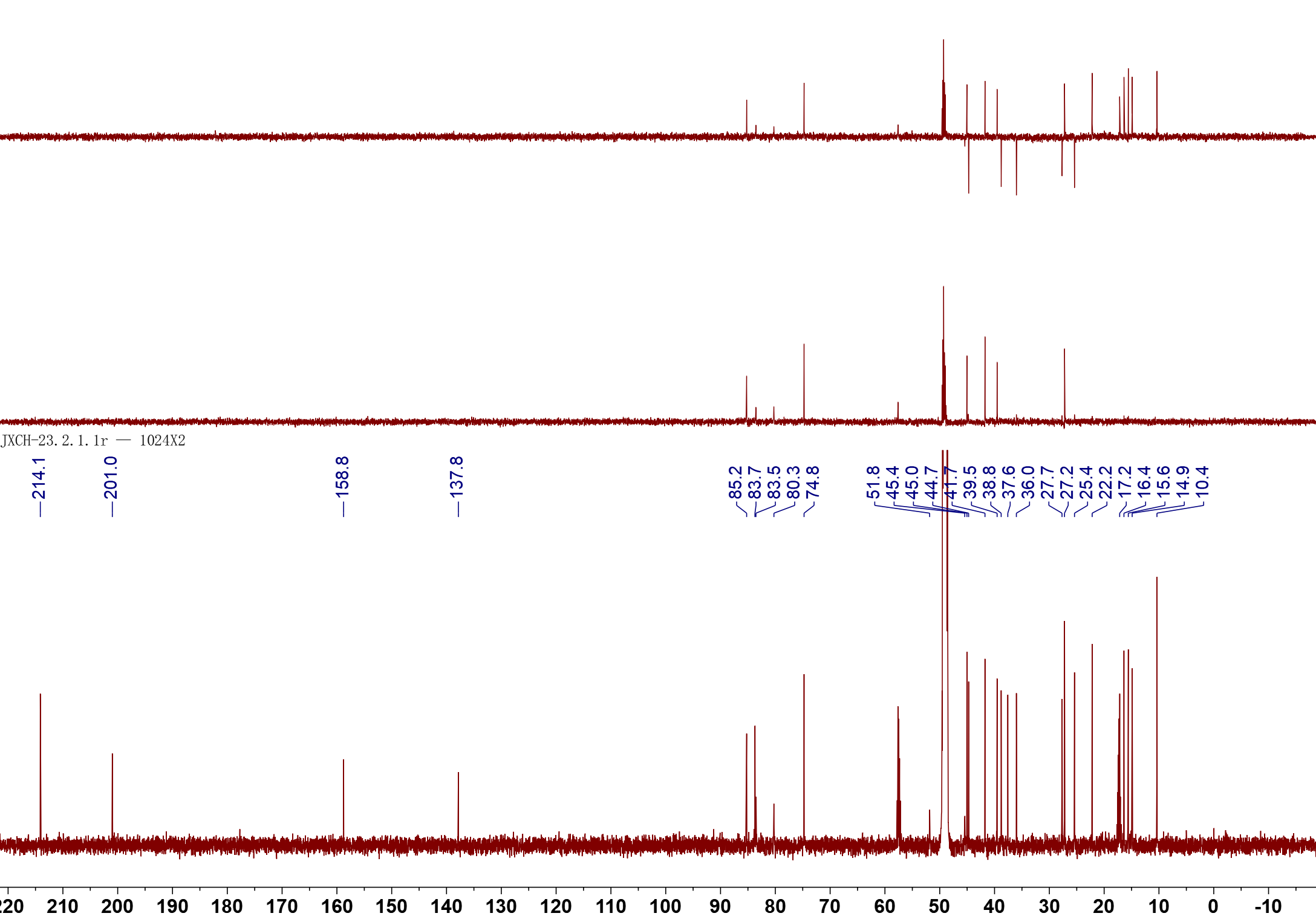


## Figure S3. HSQC spectrum of 1 (CD_3_OD).


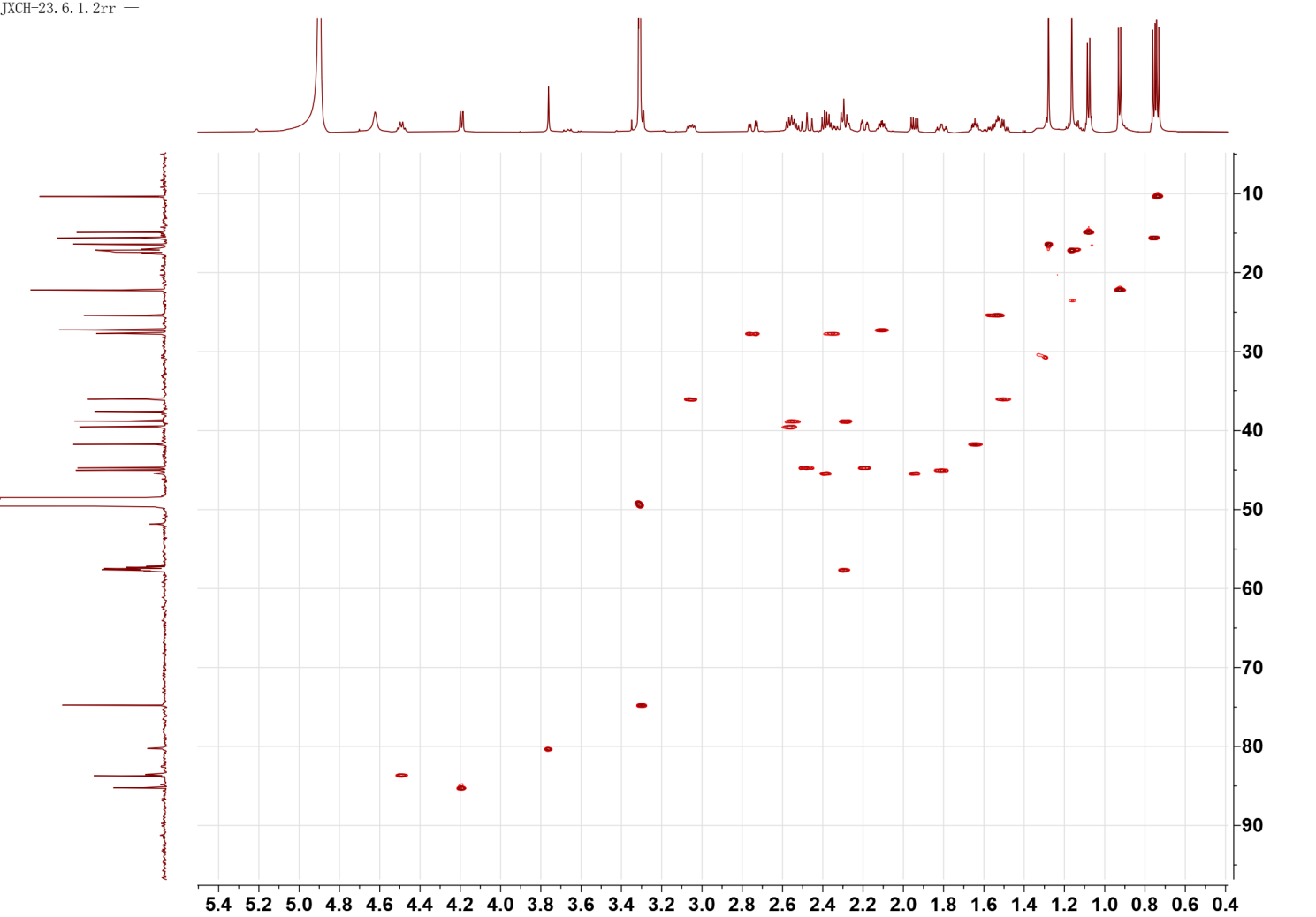


## Figure S4. ^1^H-^1^H COSY spectrum of 1 (CD_3_OD).


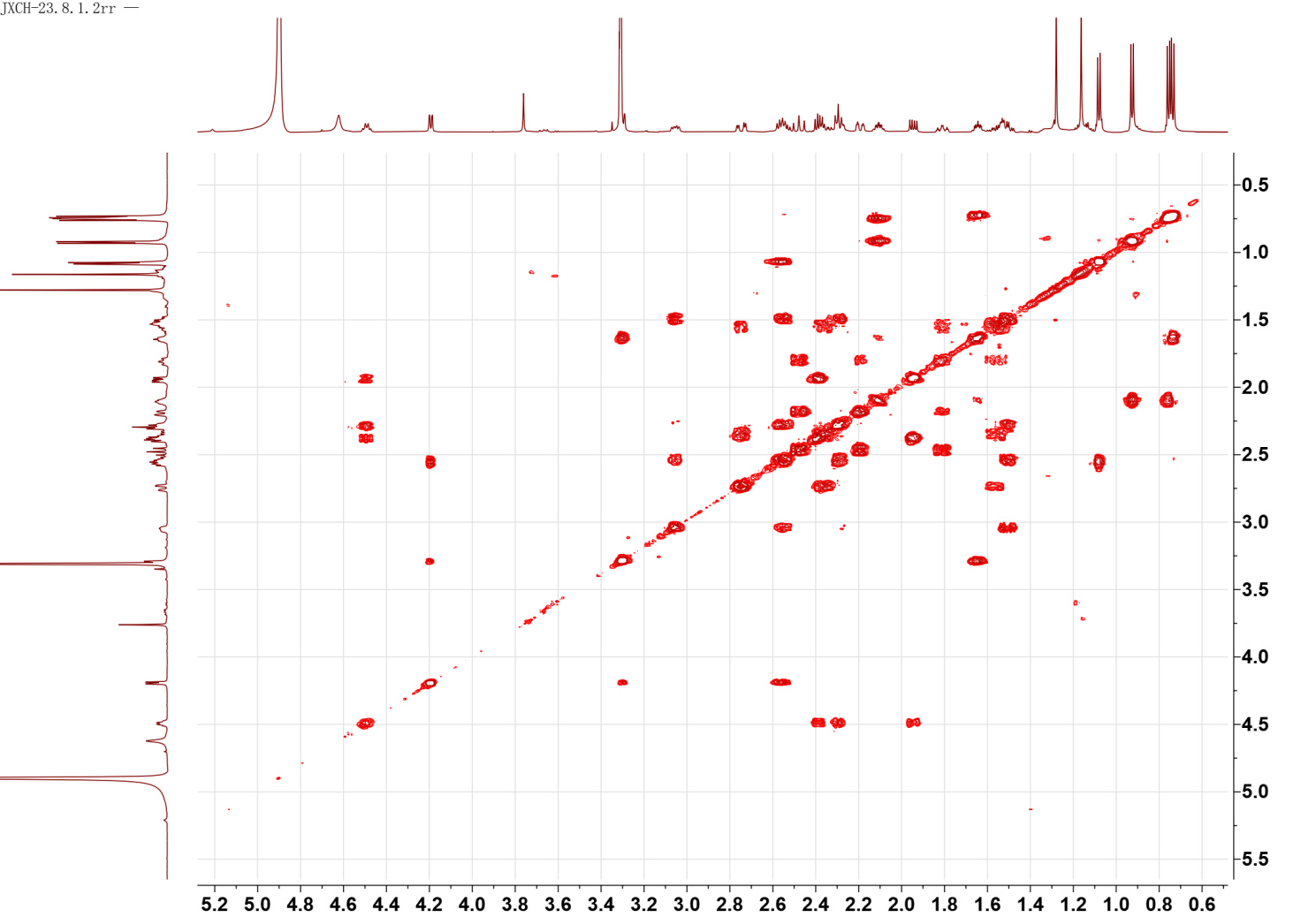


## Figure S5. HMBC spectrum of 1 (CD_3_OD).


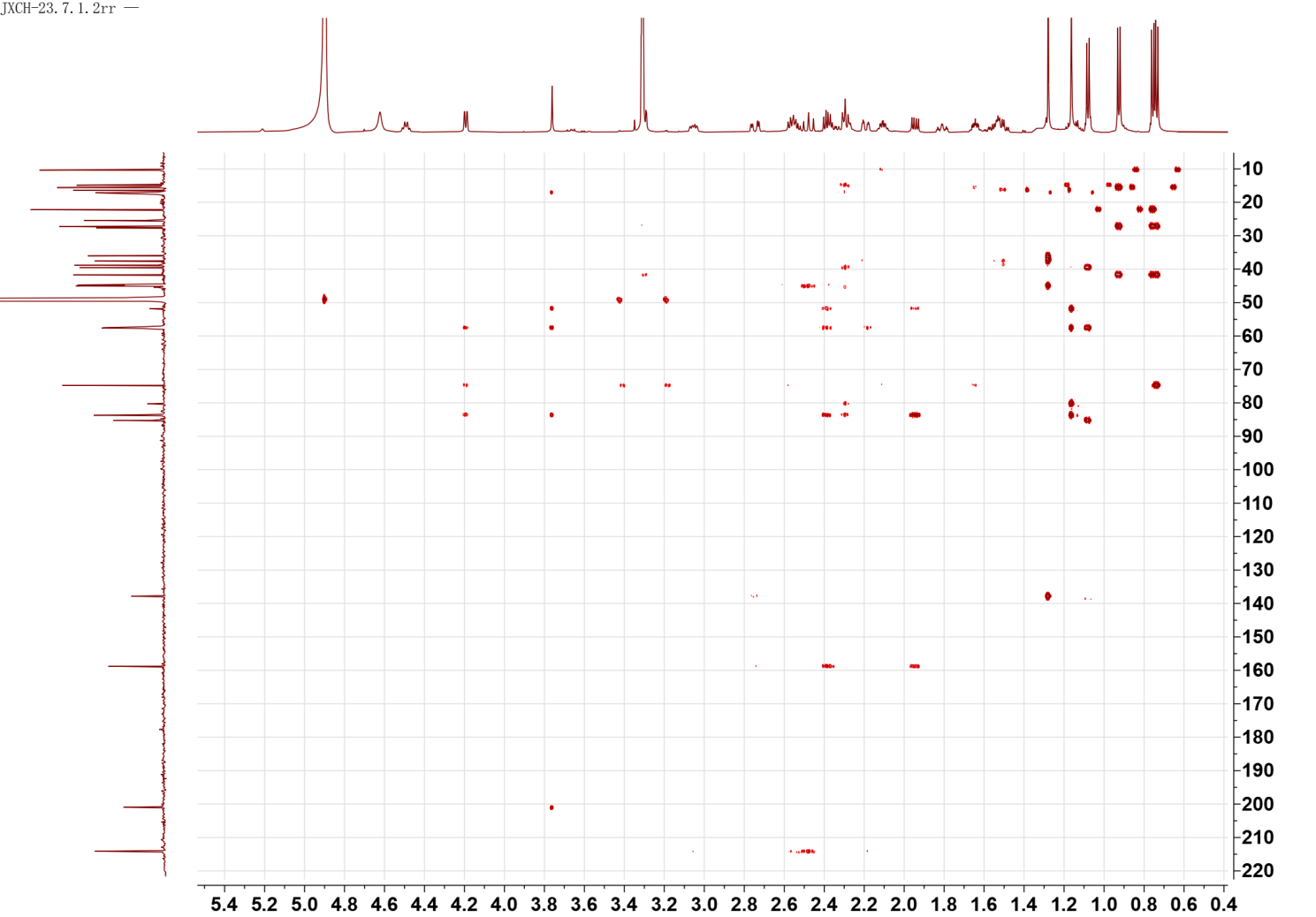


## Figure S6. HRESIMS report of 1.


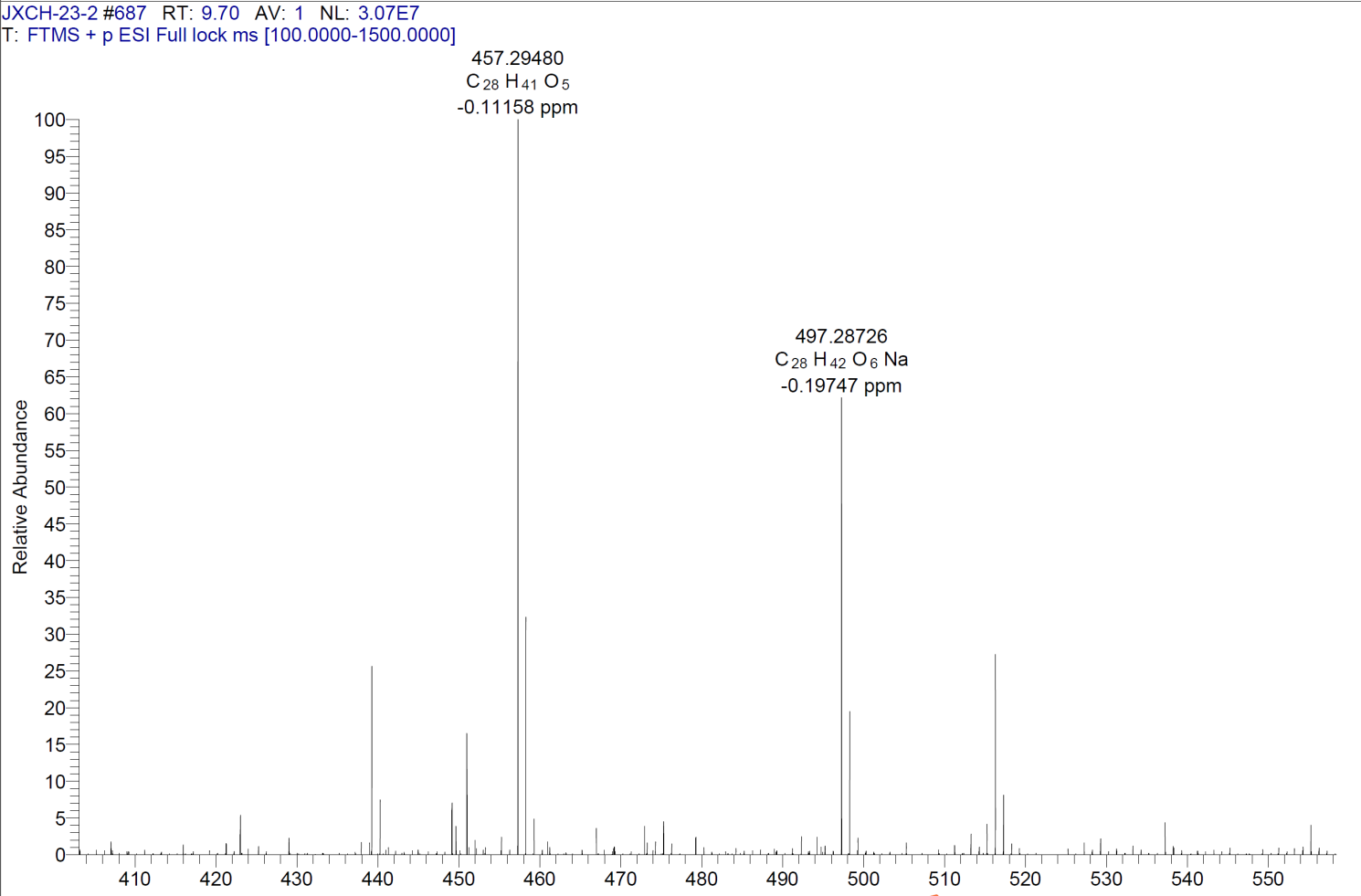


## Figure S7. ^1^H NMR spectrum of 1 (600 MHz, DMSO-*d*_6_).


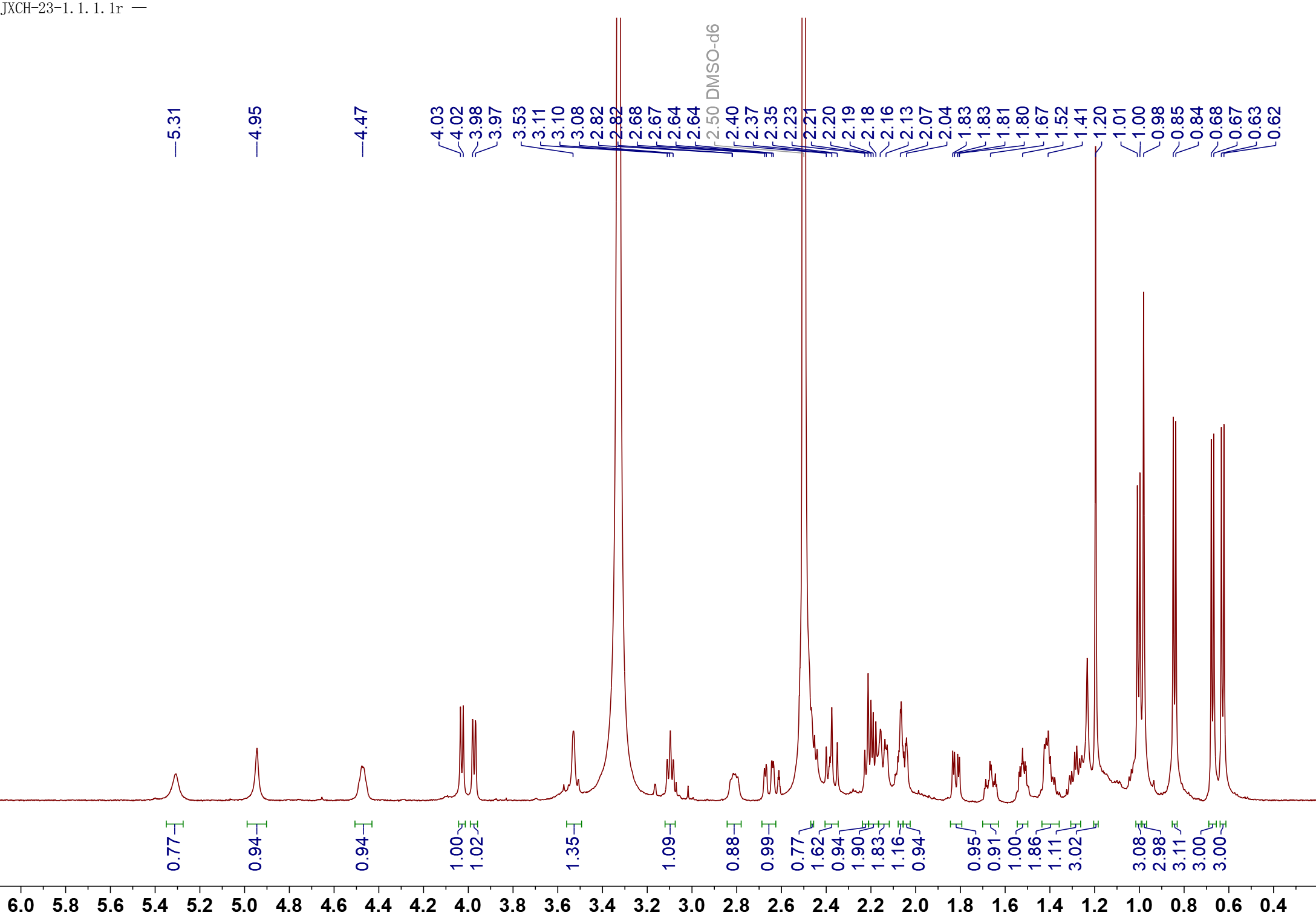


## Figure S8. ^13^C and DEPT NMR spectra of 1 (150 MHz, DMSO-*d*_6_).


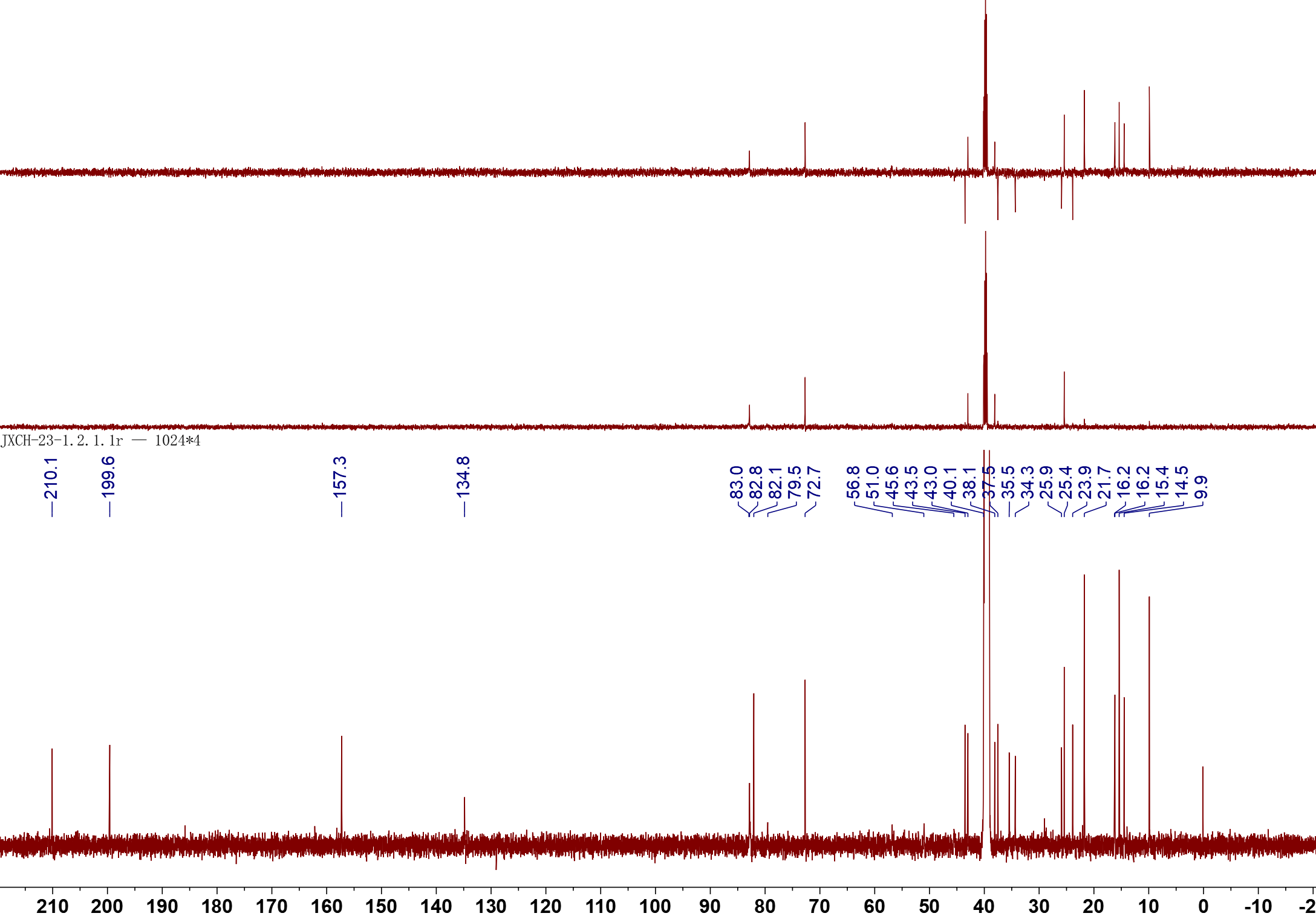


## Figure S9. HSQC spectrum of 1 (DMSO-*d*_6_).

## Figure S10. ^1^H-^1^H COSY spectrum of 1 (DMSO-*d*_6_).

## Figure S11. HMBC spectrum of 1 (DMSO-*d*_6_).


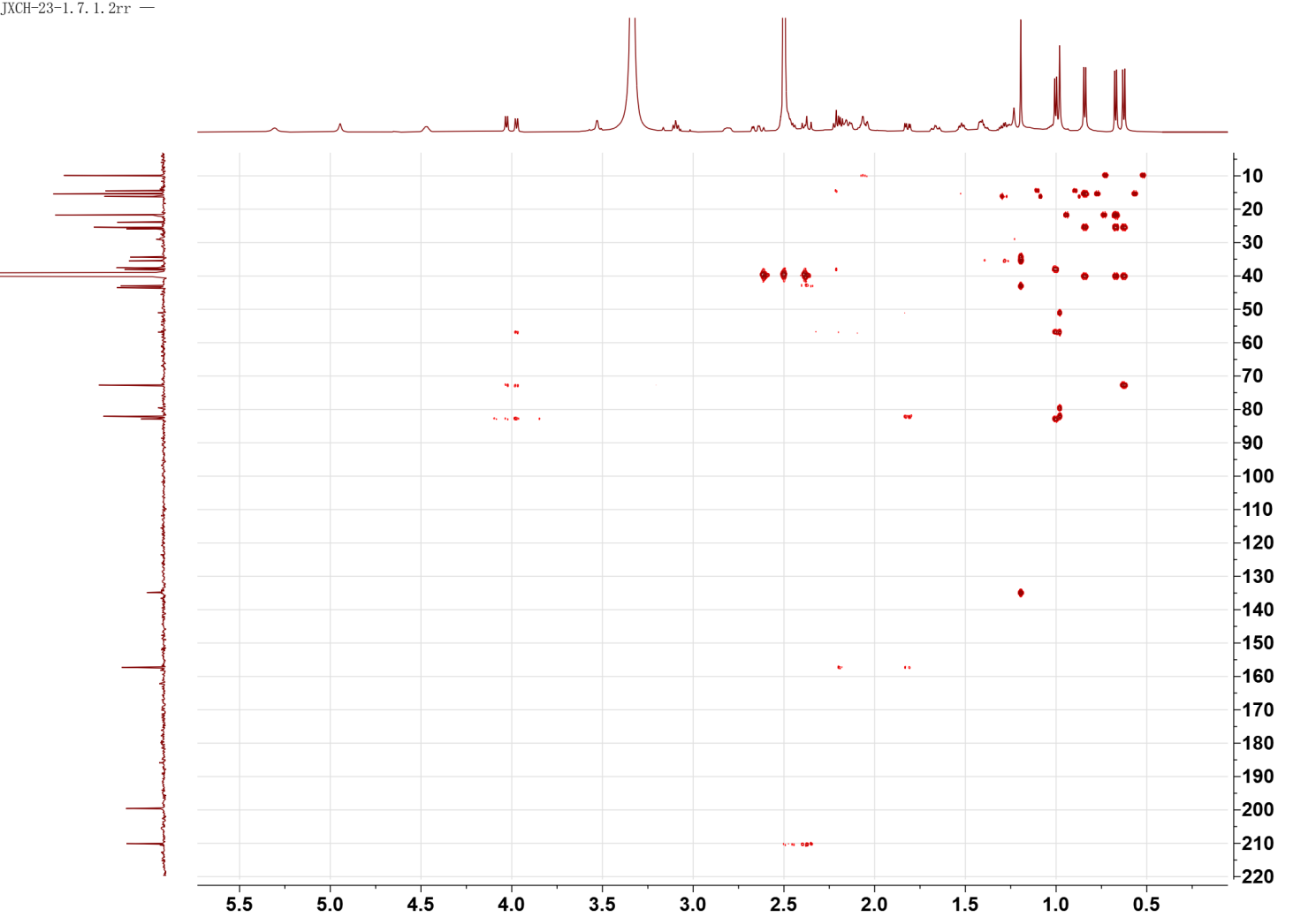


## Figure S12. ROESY spectrum of 1 (DMSO-*d*_6_).


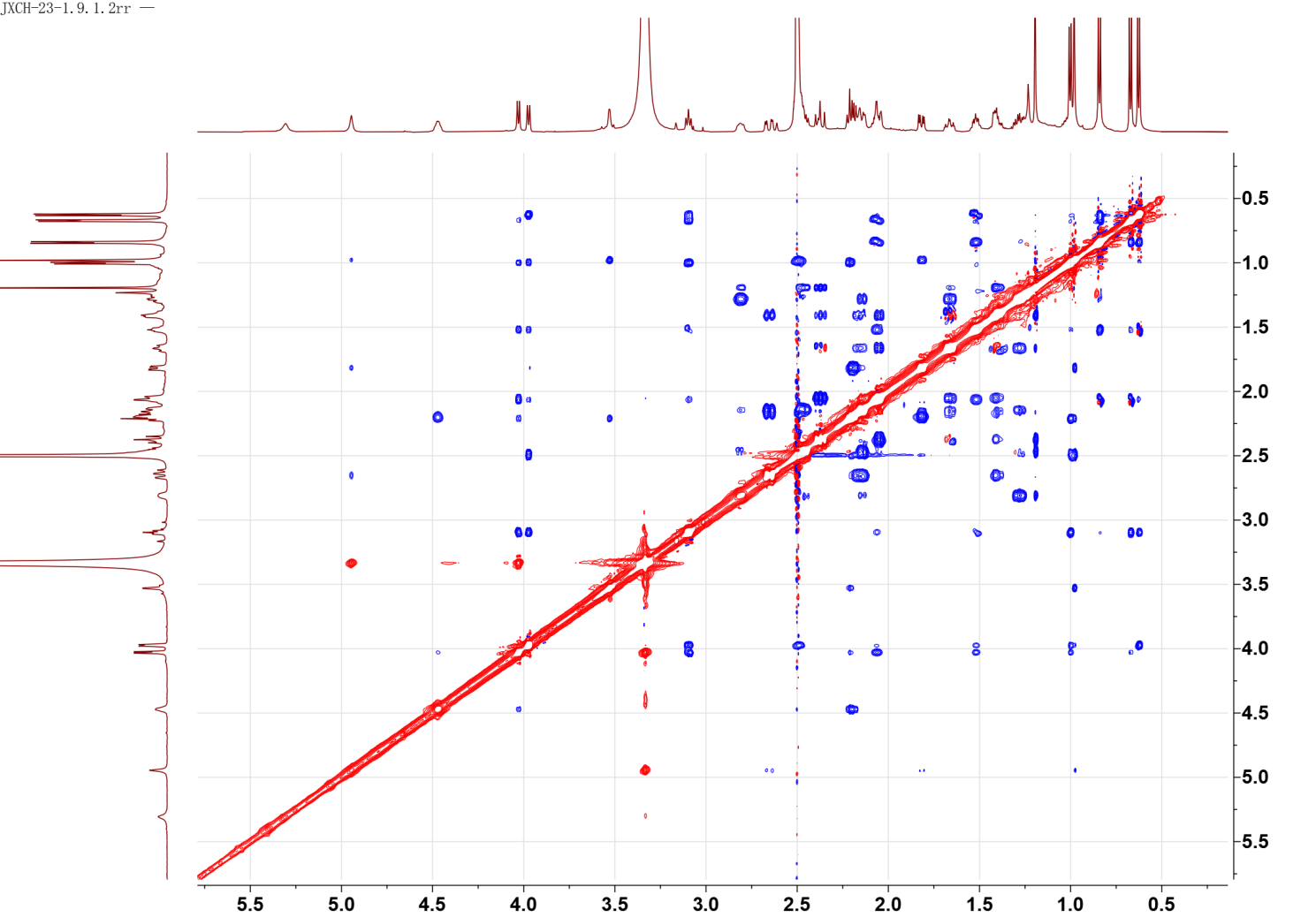


## Figure S13. ^1^H NMR spectrum of 2 (600 MHz, CD_3_OD).


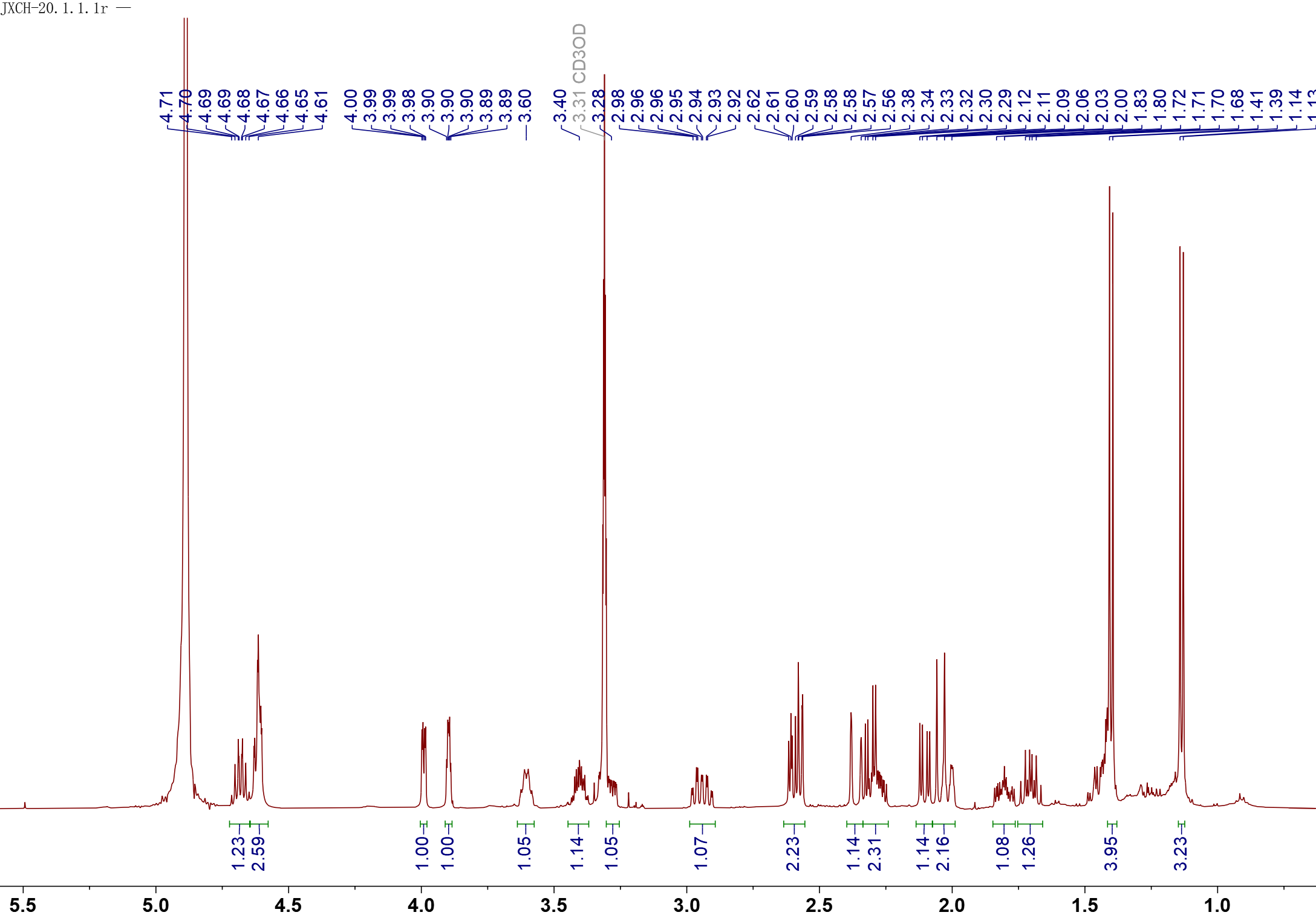


## Figure S14. ^13^C and DEPT NMR spectra of 2 (150 MHz, CD_3_OD).


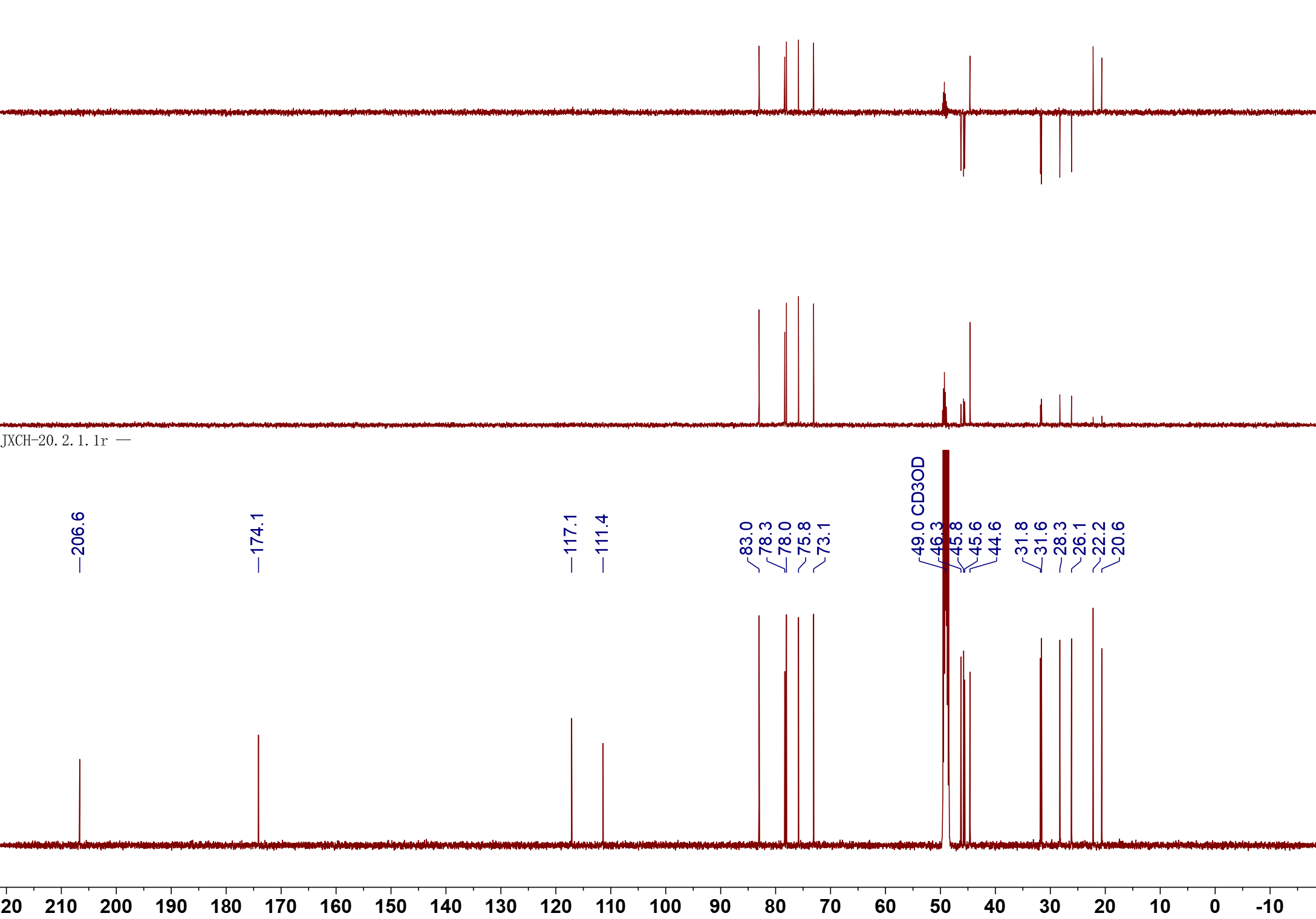


## Figure S15. HSQC spectrum of 2 (CD_3_OD).


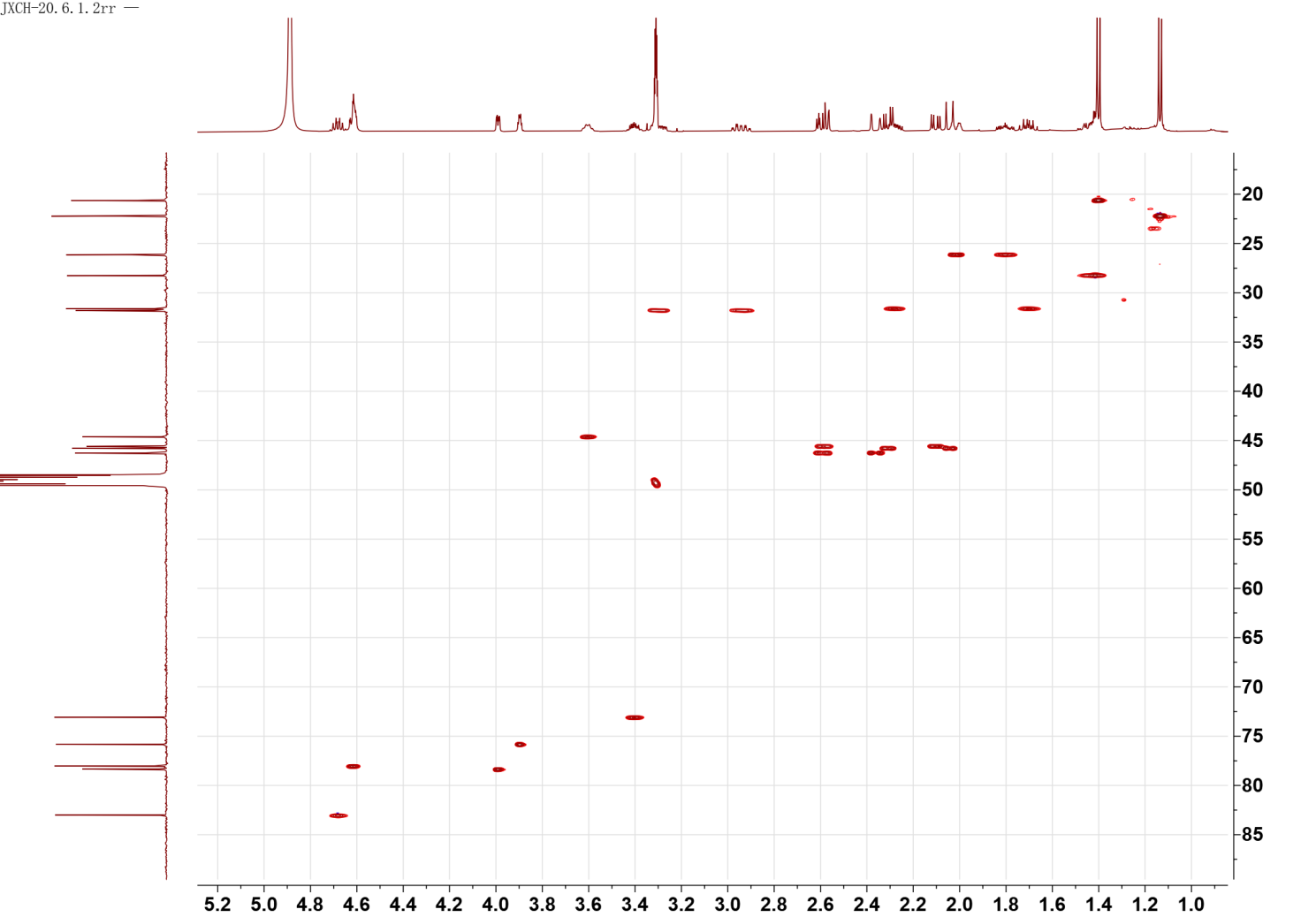


## Figure S16. ^1^H-^1^H COSY spectrum of 2 (CD_3_OD).


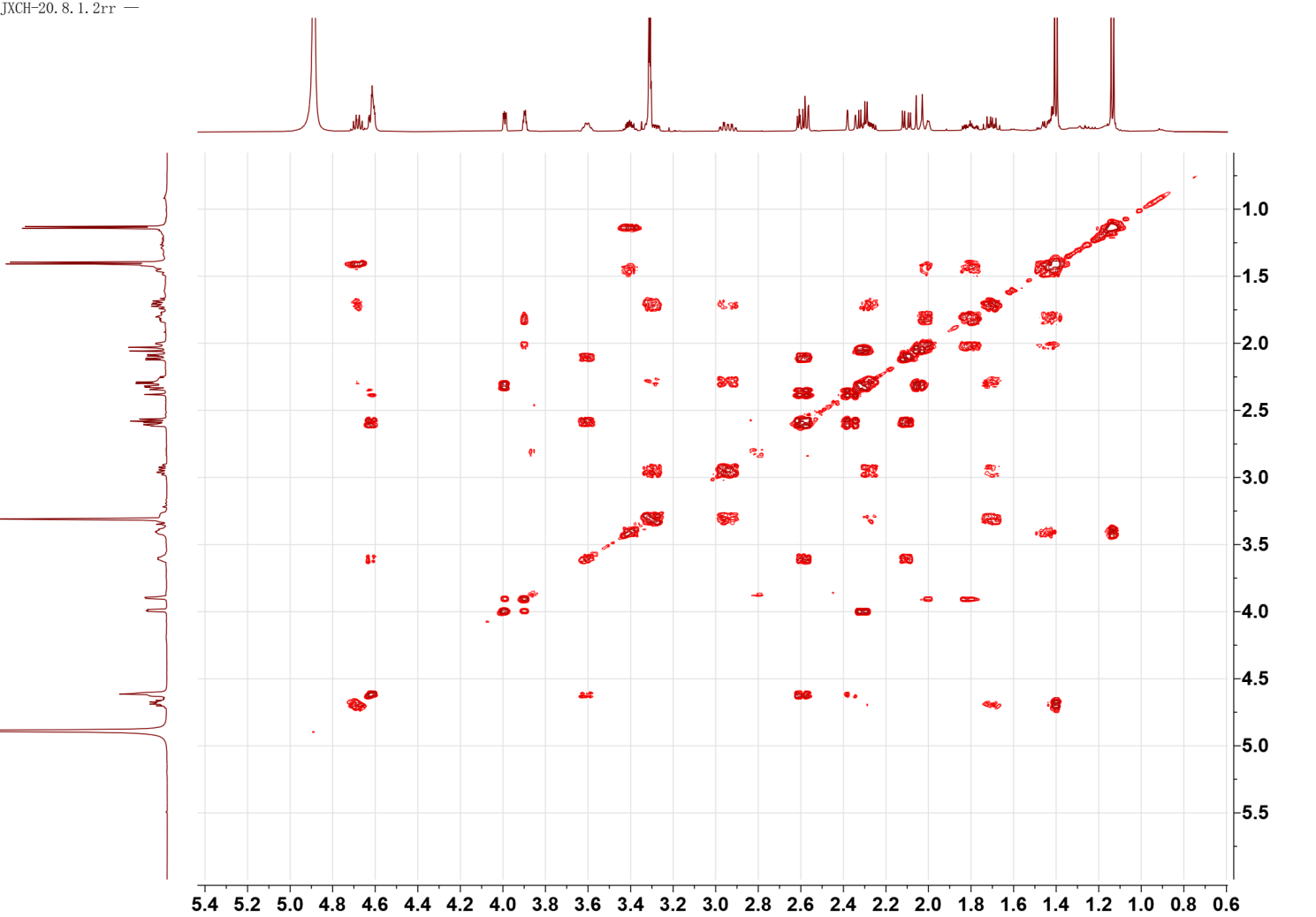


## Figure S17. HMBC spectrum of 2 (CD_3_OD).


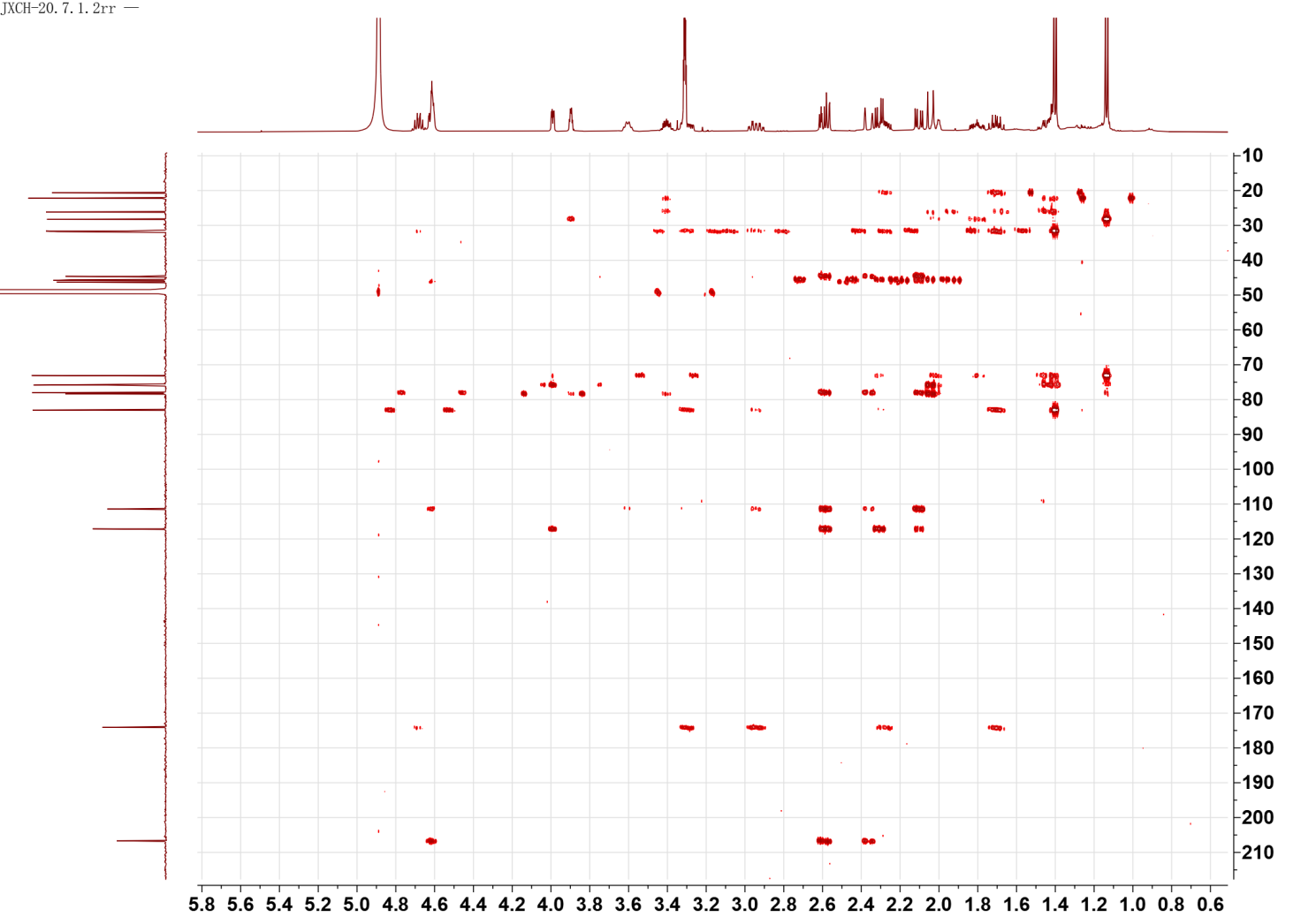


## Figure S18. ROESY spectrum of 2 (CD_3_OD).


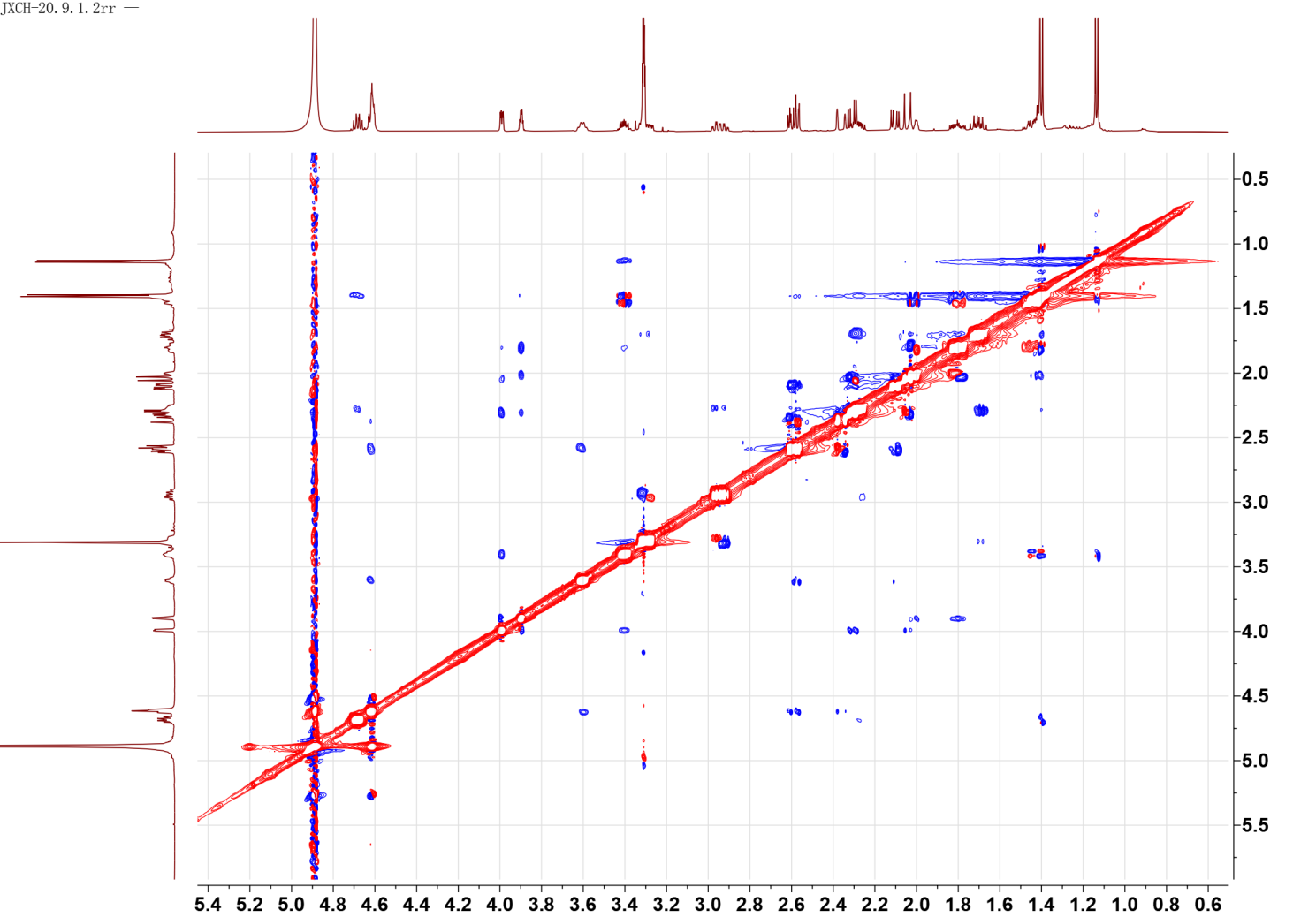


## Figure S19. HRESIMS report of 2.


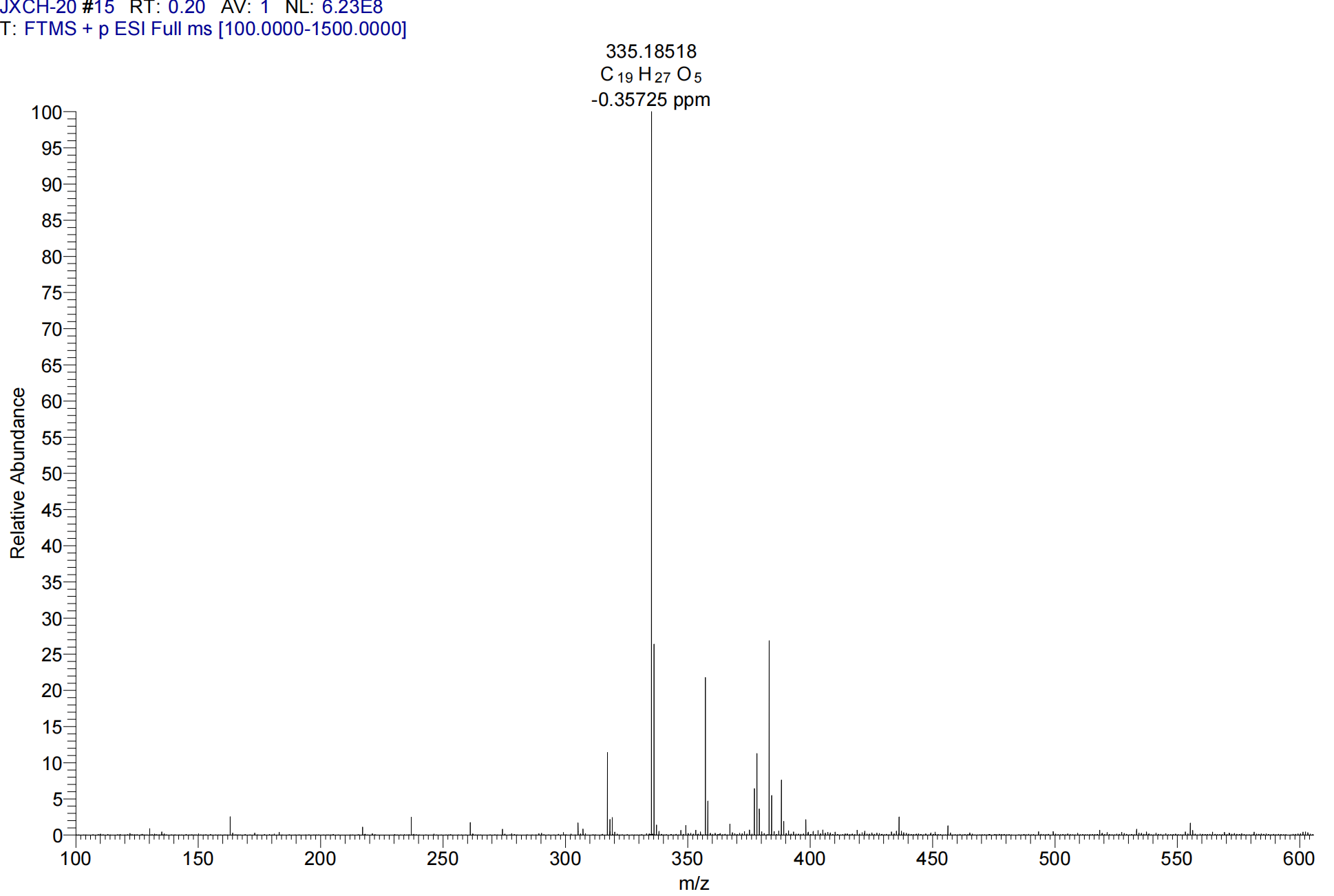


## Figure S20. ^1^H NMR spectrum of 3 (600 MHz, CD_3_COCD_3_).


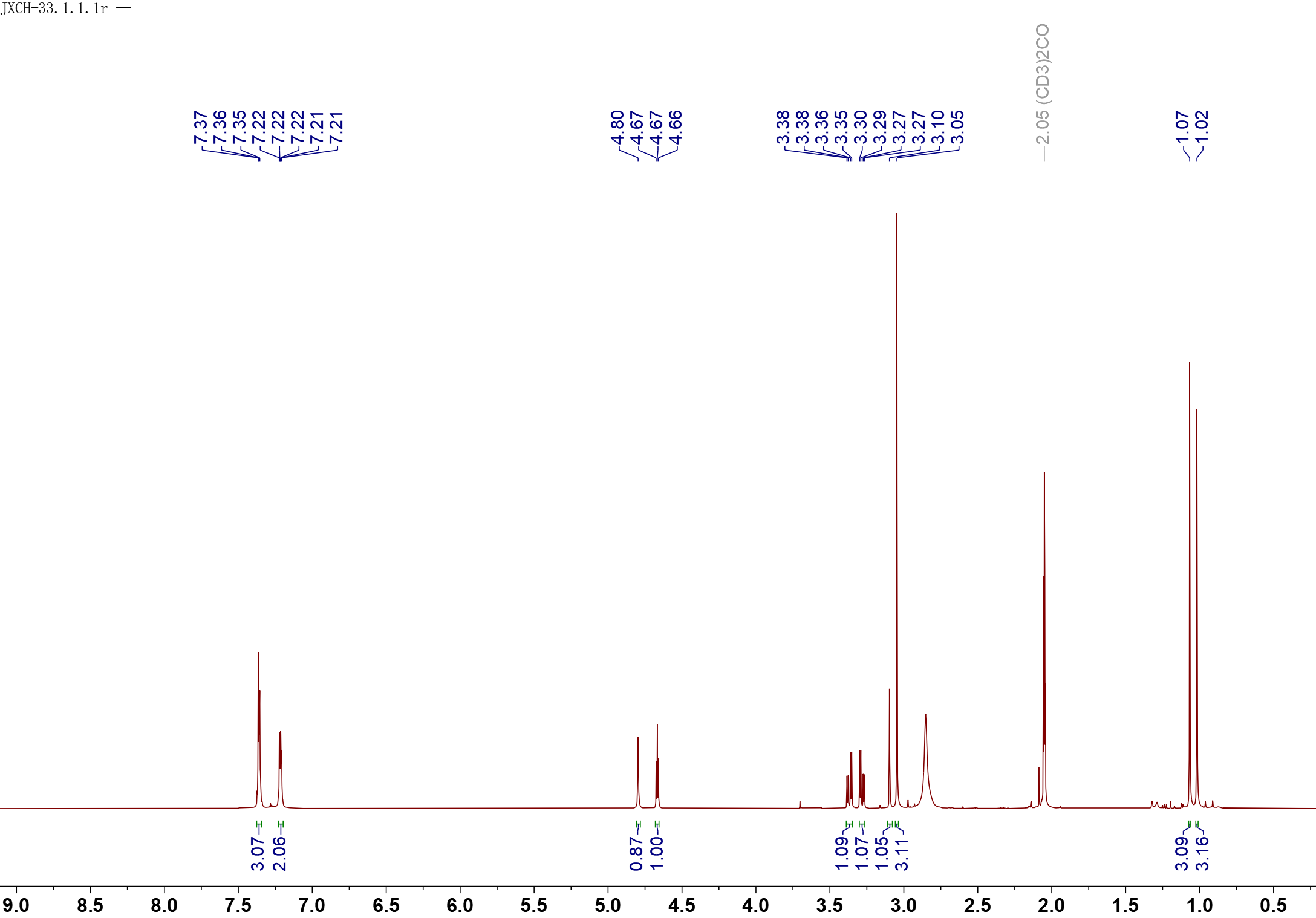


## Figure S21. ^13^C and DEPT NMR spectra of 3 (150 MHz, CD_3_COCD_3_).


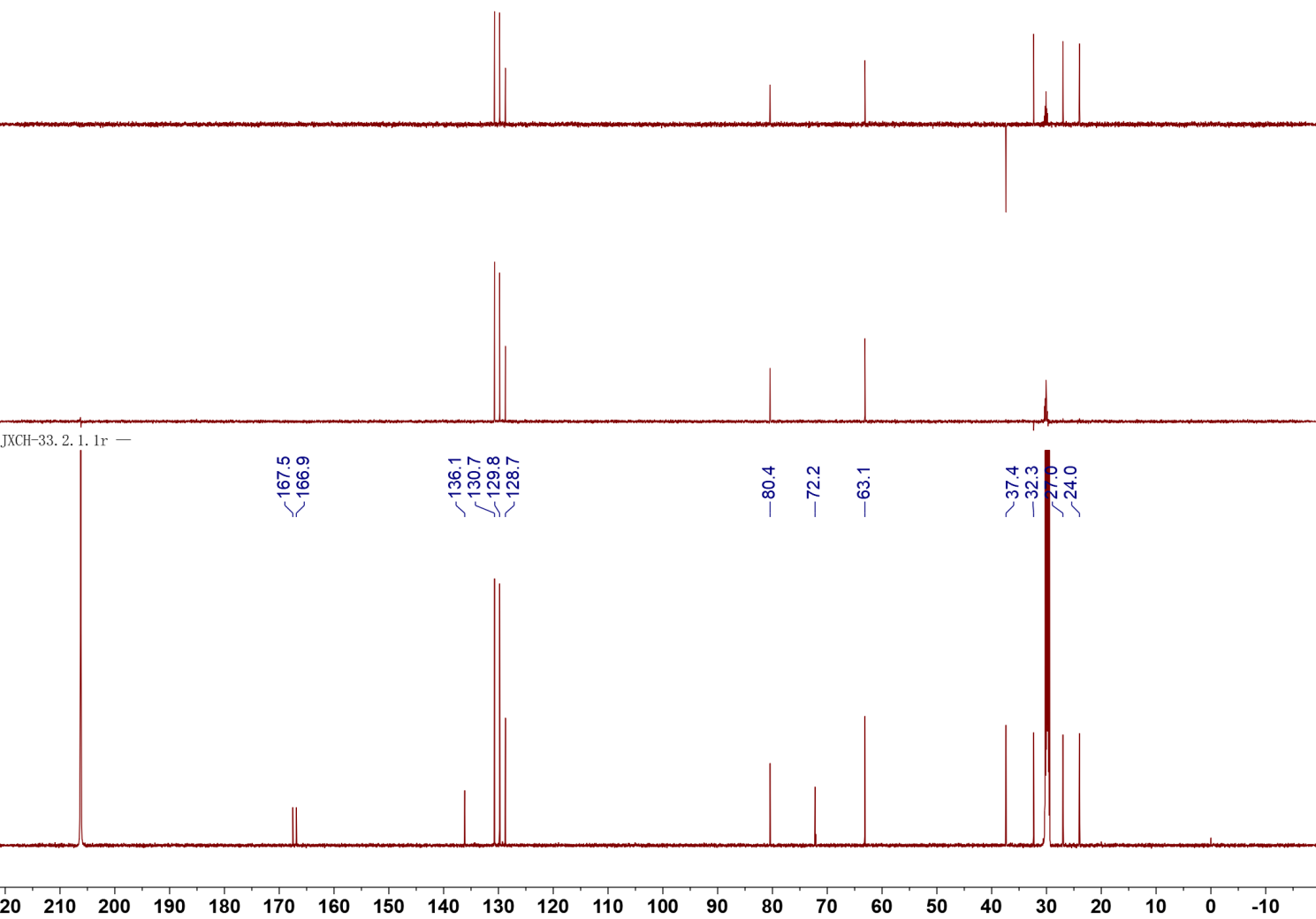


## Figure S22. HSQC spectrum of 3 (CD_3_COCD_3_).


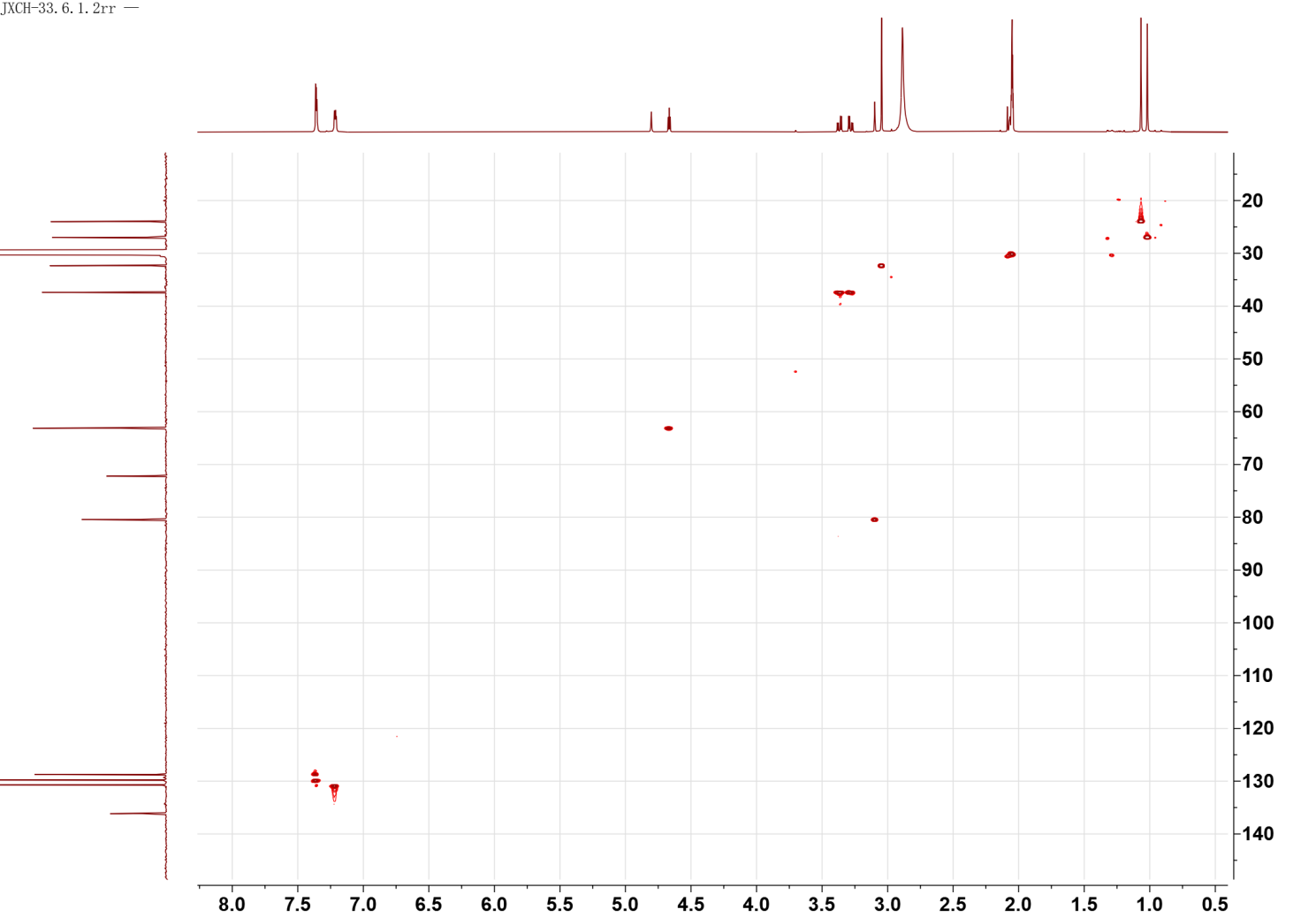


## Figure S23. ^1^H-^1^H COSY spectrum of 3 (CD_3_COCD_3_).


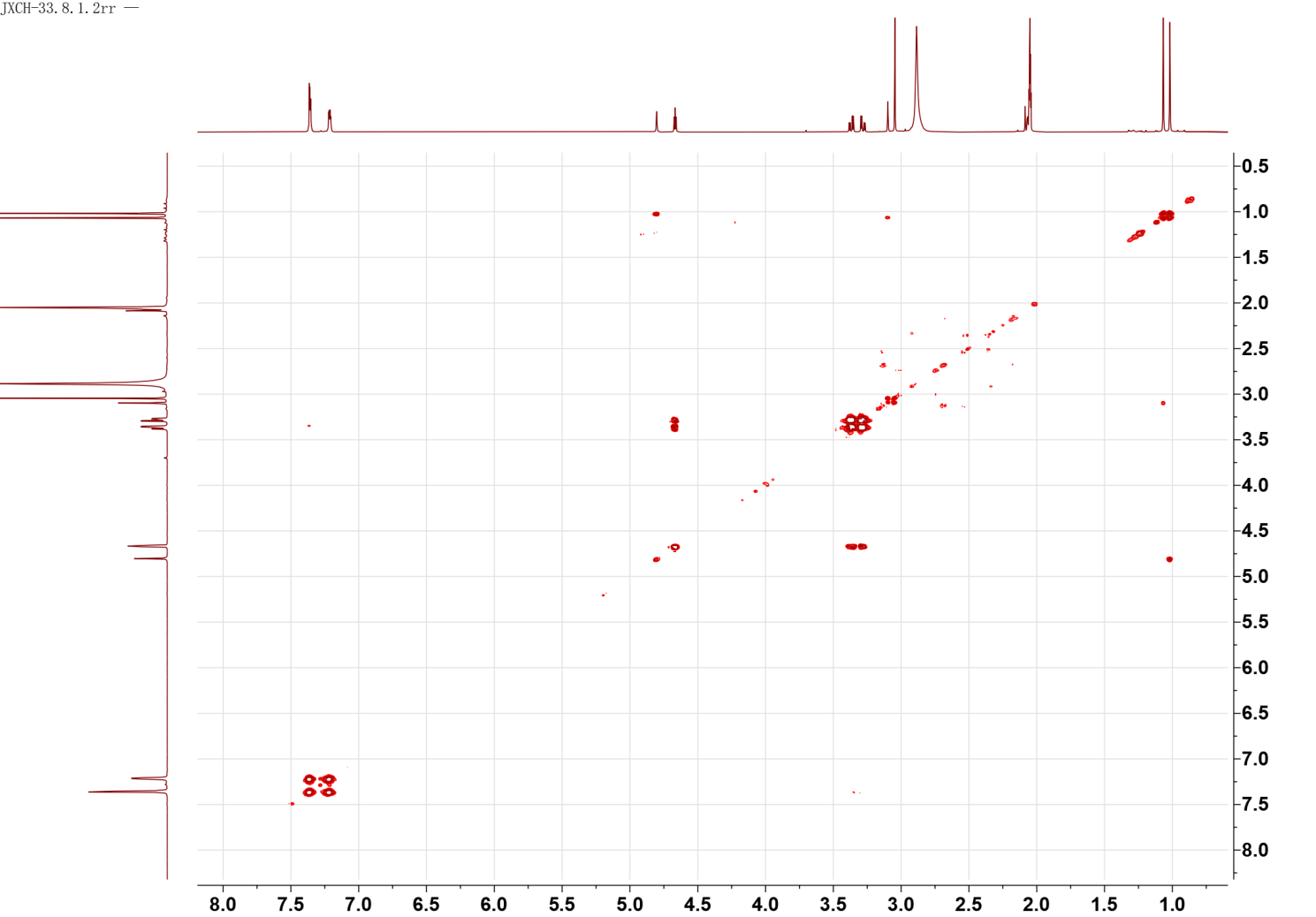


## Figure S24. HMBC spectrum of 3 (CD_3_COCD_3_).


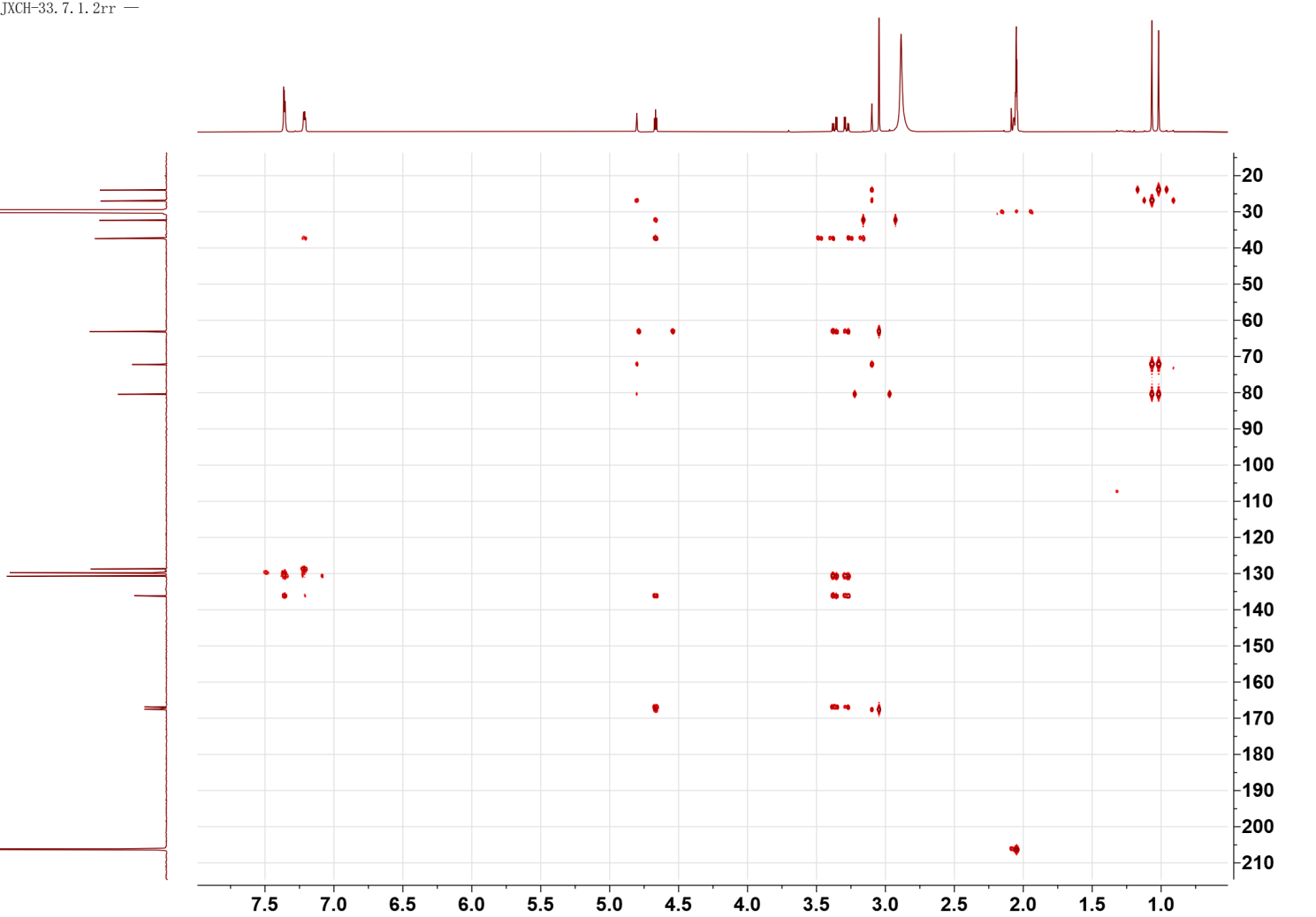


## Figure S25. ROESY spectrum of 3 (CD_3_COCD_3_).


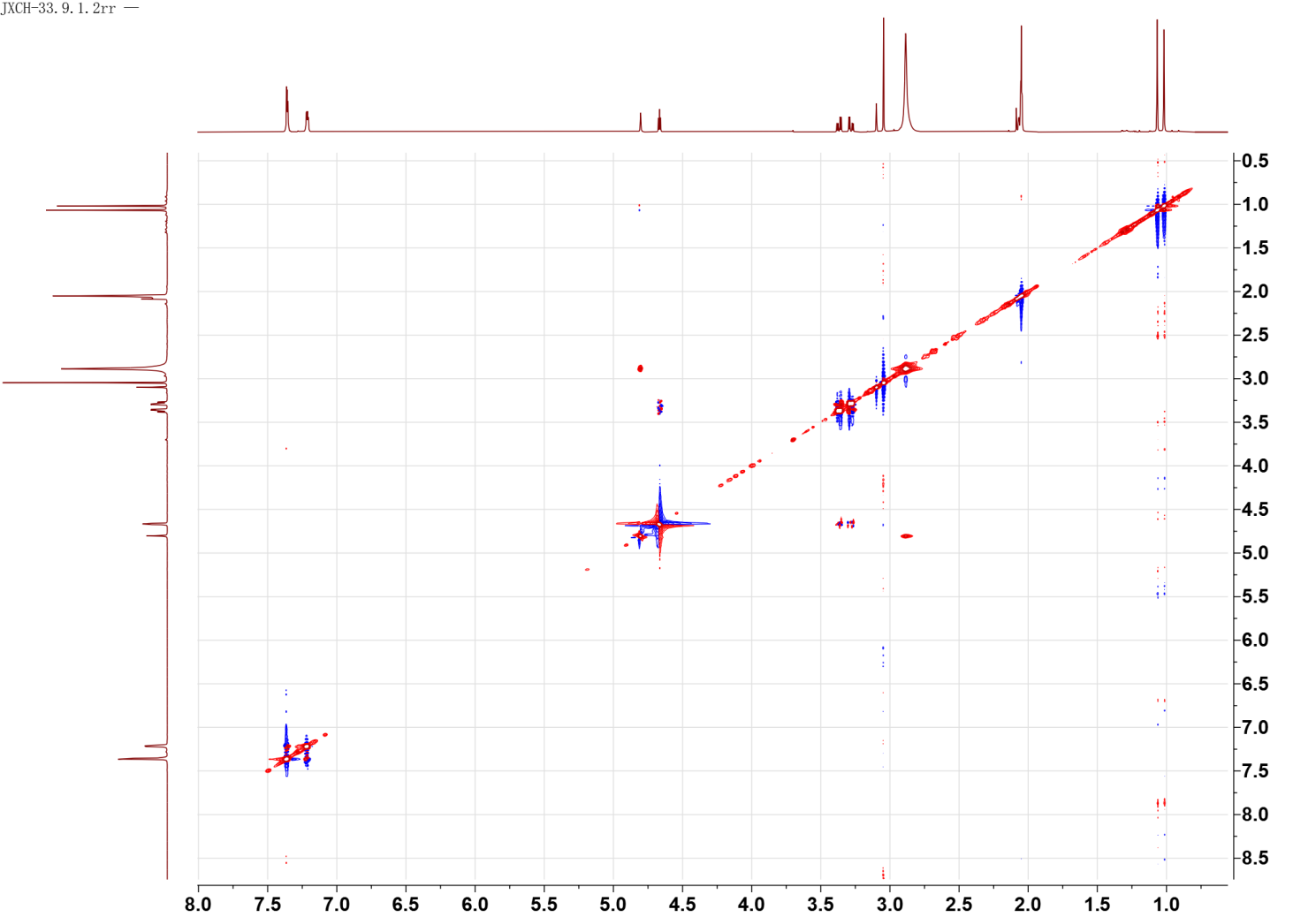


## Figure S26 HRESIMS report of 3.


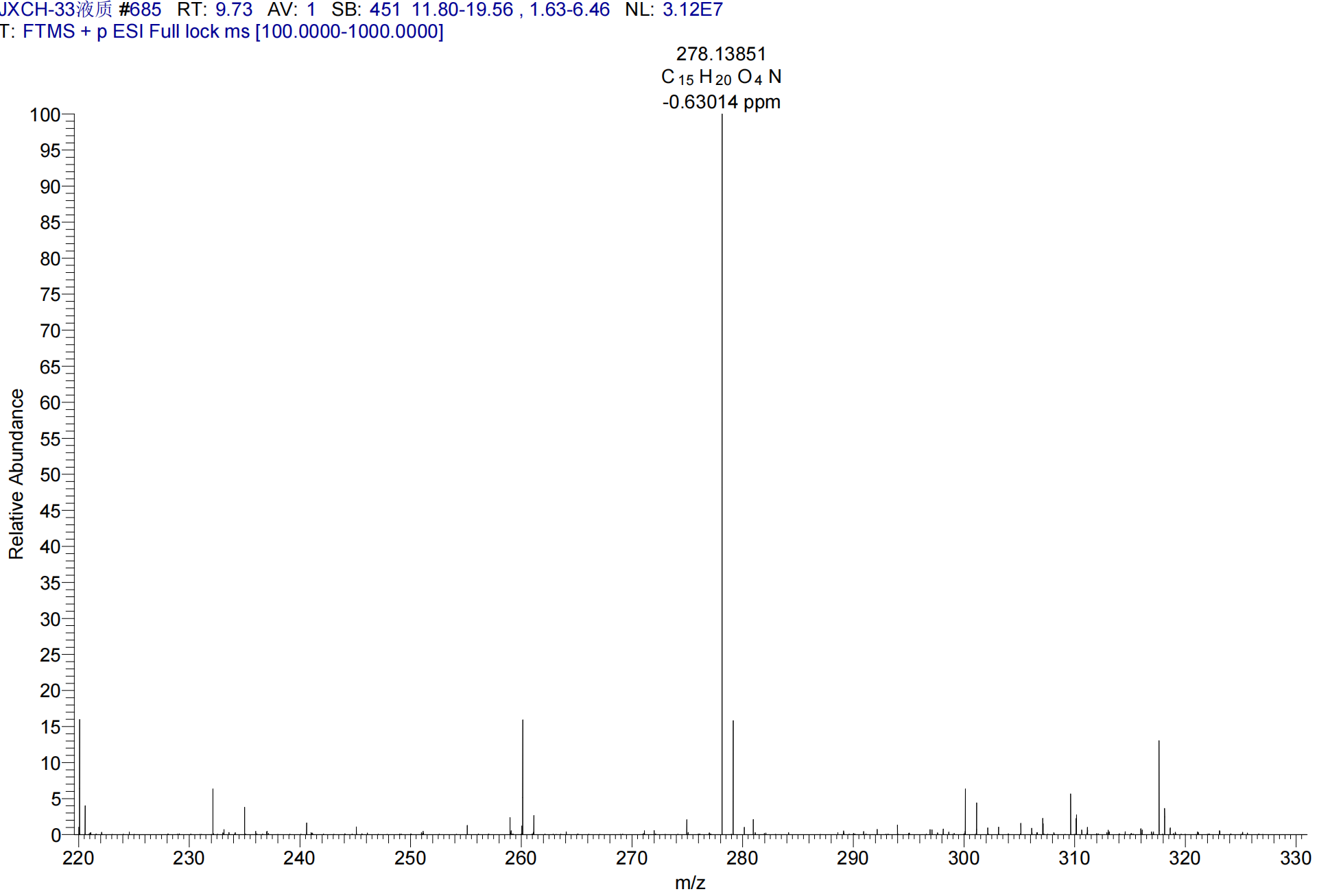


## Figure S27. CD spectrum of 1.


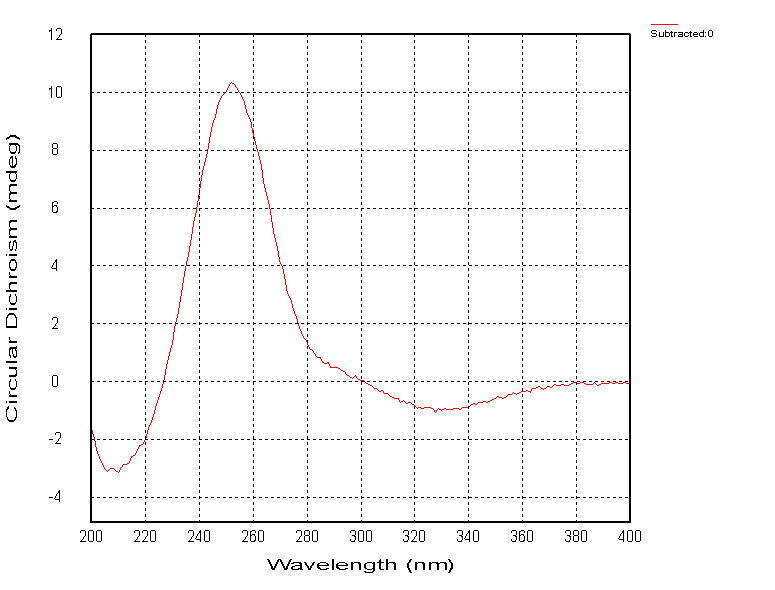


## Figure S28. CD spectrum of 2.


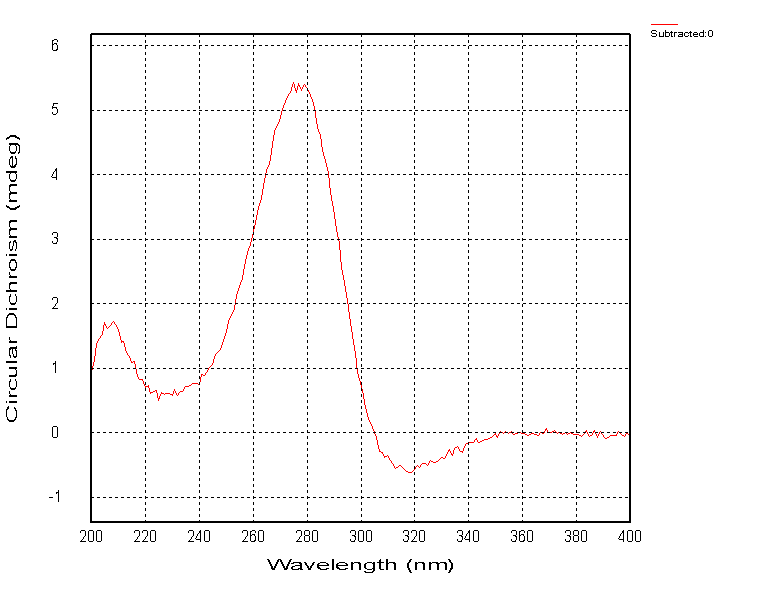


## Figure S29. CD spectrum of 3.


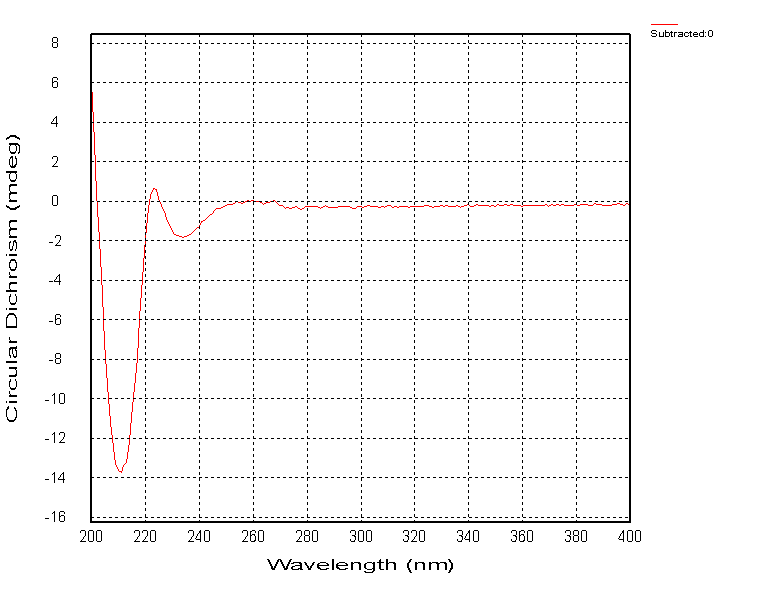


# Calculation details.

## 2.1 ECD calculation of 1.

Conformation search of **1** at MMFF94s force field gave six conformers **1a**-**1f**. These conformers were optimized at B3LYP/6-31G(d) level, and then calculated the ECD at B3LYP/6-31G(d,p) level.

| Conformers | Gibbs free energies | Population (%) |
| --- | --- | --- |
| **1a** | －1542.979459 | 7.3 |
| **1b** | －1542.979462 | 7.4 |
| **1c** | －1542.981013 | 38.1 |
| **1d** | －1542.979221 | 5.7 |
| **1e** | －1542.981015 | 38.2 |
| **1f** | －1542.978697 | 3.3 |

Standard orientation of optimized **1** at B3LYP/6-31G(d) level.

**1a**

| C | 5.450625 | 1.35028 | -1.719166 |
| --- | --- | --- | --- |
| C | 6.07685 | 1.930423 | -0.458647 |
| C | 5.887688 | 1.104923 | 0.808702 |
| C | 4.433892 | 0.614965 | 0.939844 |
| C | 3.971045 | -0.220987 | -0.289685 |
| C | 4.04475 | 0.744301 | -1.511863 |
| C | 4.131732 | -0.103123 | 2.253225 |
| C | 2.614698 | -0.201423 | 2.40938 |
| C | 1.903666 | -0.665643 | 1.153801 |
| C | 2.528871 | -0.724185 | -0.053407 |
| C | 0.439436 | -1.08323 | 1.397766 |
| C | -0.355121 | -1.482206 | 0.096539 |
| C | 0.580944 | -2.218541 | -0.864167 |
| C | 1.832629 | -1.400887 | -1.173392 |
| C | -0.388665 | 0.114575 | 1.926026 |
| C | -0.725331 | 0.934025 | 0.671271 |
| C | -0.806142 | -0.101626 | -0.496276 |
| C | -1.51449 | -2.437713 | 0.437234 |
| C | 4.863105 | -1.466495 | -0.534078 |
| O | 6.687388 | 2.982619 | -0.459306 |
| O | -0.082741 | -2.545918 | -2.063229 |
| O | 0.544911 | -2.15003 | 2.346253 |
| O | 2.238183 | -1.419775 | -2.334996 |
| O | -1.980789 | 1.605455 | 0.73161 |
| C | -2.975357 | 0.743877 | 0.139871 |
| C | -2.248499 | 0.081587 | -1.05247 |
| C | -2.216036 | 0.933981 | -2.331206 |
| C | -4.206784 | 1.624918 | -0.099698 |
| O | -4.556365 | 2.256725 | 1.132946 |
| C | -5.445575 | 0.905655 | -0.678697 |
| C | -6.544997 | 1.947418 | -0.955476 |
| C | -5.952958 | -0.330565 | 0.124201 |
| C | -6.672255 | 0.011185 | 1.439945 |
| C | -6.841373 | -1.229741 | -0.75429 |
| H | 5.430056 | 2.134352 | -2.482225 |
| H | 6.134147 | 0.571521 | -2.088904 |
| H | 6.583051 | 0.253756 | 0.785564 |
| H | 6.169988 | 1.726895 | 1.66443 |
| H | 3.80646 | 1.521878 | 0.938335 |
| H | 3.73908 | 0.229488 | -2.422123 |
| H | 3.32622 | 1.559711 | -1.350437 |
| H | 4.587535 | -1.100967 | 2.2665 |
| H | 4.555503 | 0.447098 | 3.102073 |
| H | 2.334884 | -0.88256 | 3.21979 |
| H | 2.227052 | 0.785841 | 2.699948 |
| H | 0.938051 | -3.134809 | -0.357426 |
| H | -1.321098 | -0.273066 | 2.357131 |
| H | 0.108217 | 0.690228 | 2.709316 |
| H | 0.032841 | 1.704205 | 0.496804 |
| H | -0.105222 | 0.175688 | -1.288523 |
| H | -2.262404 | -1.990628 | 1.101151 |
| H | -2.020946 | -2.749791 | -0.478269 |
| H | -1.126456 | -3.343087 | 0.914844 |
| H | 5.906746 | -1.199883 | -0.723851 |
| H | 4.846499 | -2.145939 | 0.324218 |
| H | 4.504647 | -2.018572 | -1.406662 |
| H | 0.585628 | -2.396587 | -2.762653 |
| H | -0.338582 | -2.305153 | 2.715455 |
| H | -3.245446 | -0.026201 | 0.877184 |
| H | -2.709267 | -0.880803 | -1.288362 |
| H | -1.605918 | 0.432226 | -3.090141 |
| H | -3.214244 | 1.083656 | -2.754863 |
| H | -1.77849 | 1.921021 | -2.142262 |
| H | -3.914183 | 2.400586 | -0.825997 |
| H | -3.723305 | 2.606363 | 1.49153 |
| H | -5.11779 | 0.514162 | -1.653454 |
| H | -7.431832 | 1.488418 | -1.404263 |
| H | -6.180798 | 2.710988 | -1.653205 |
| H | -6.845824 | 2.457694 | -0.036896 |
| H | -5.070311 | -0.932954 | 0.382512 |
| H | -7.610292 | 0.547283 | 1.252353 |
| H | -6.926524 | -0.908837 | 1.981006 |
| H | -6.051283 | 0.639195 | 2.082094 |
| H | -7.77292 | -0.726688 | -1.039424 |
| H | -7.118376 | -2.142818 | -0.214093 |
| H | -6.32787 | -1.530004 | -1.67614 |

**1b**

| C | 5.450102 | 1.351418 | -1.718877 |
| --- | --- | --- | --- |
| C | 6.07576 | 1.931599 | -0.458106 |
| C | 5.887589 | 1.105091 | 0.808742 |
| C | 4.433909 | 0.614665 | 0.939898 |
| C | 3.971098 | -0.220927 | -0.289914 |
| C | 4.044441 | 0.74479 | -1.511805 |
| C | 4.132017 | -0.104028 | 2.253011 |
| C | 2.615011 | -0.20254 | 2.409286 |
| C | 1.903911 | -0.666324 | 1.153614 |
| C | 2.529034 | -0.724374 | -0.05366 |
| C | 0.439713 | -1.084022 | 1.397512 |
| C | -0.354917 | -1.482586 | 0.096203 |
| C | 0.581148 | -2.218545 | -0.864802 |
| C | 1.832757 | -1.400695 | -1.17383 |
| C | -0.388335 | 0.113638 | 1.926211 |
| C | -0.725186 | 0.933446 | 0.671753 |
| C | -0.806082 | -0.10182 | -0.496111 |
| C | -1.514221 | -2.438281 | 0.436655 |
| C | 4.863367 | -1.46615 | -0.534873 |
| O | 6.684904 | 2.984589 | -0.458165 |
| O | -0.082559 | -2.545614 | -2.063938 |
| O | 0.545262 | -2.151091 | 2.345647 |
| O | 2.238296 | -1.41923 | -2.335441 |
| O | -1.980666 | 1.604811 | 0.732473 |
| C | -2.975284 | 0.743597 | 0.140327 |
| C | -2.248471 | 0.081593 | -1.052196 |
| C | -2.215981 | 0.934165 | -2.330804 |
| C | -4.206538 | 1.624944 | -0.099119 |
| O | -4.556133 | 2.256508 | 1.133623 |
| C | -5.445411 | 0.906085 | -0.67844 |
| C | -6.544615 | 1.948141 | -0.955028 |
| C | -5.953081 | -0.330266 | 0.124037 |
| C | -6.672498 | 0.011173 | 1.439798 |
| C | -6.841529 | -1.229054 | -0.754825 |
| H | 5.429244 | 2.135631 | -2.481773 |
| H | 6.133965 | 0.572972 | -2.088647 |
| H | 6.583136 | 0.254114 | 0.78474 |
| H | 6.169928 | 1.726548 | 1.664821 |
| H | 3.806264 | 1.521433 | 0.938814 |
| H | 3.739003 | 0.230099 | -2.422209 |
| H | 3.325608 | 1.559885 | -1.350141 |
| H | 4.587883 | -1.101848 | 2.265783 |
| H | 4.555846 | 0.445859 | 3.102048 |
| H | 2.335324 | -0.884002 | 3.219477 |
| H | 2.227312 | 0.784576 | 2.700295 |
| H | 0.938284 | -3.134954 | -0.358365 |
| H | -1.320705 | -0.274148 | 2.357336 |
| H | 0.108645 | 0.689074 | 2.709591 |
| H | 0.032917 | 1.703738 | 0.497446 |
| H | -0.105229 | 0.175781 | -1.288311 |
| H | -2.262135 | -1.991386 | 1.100689 |
| H | -2.020655 | -2.750116 | -0.478947 |
| H | -1.126179 | -3.343806 | 0.913979 |
| H | 5.906857 | -1.199221 | -0.725027 |
| H | 4.847317 | -2.145757 | 0.323305 |
| H | 4.504744 | -2.018193 | -1.407412 |
| H | 0.58574 | -2.395932 | -2.763349 |
| H | -0.338309 | -2.306765 | 2.714425 |
| H | -3.245552 | -0.026741 | 0.877317 |
| H | -2.70933 | -0.88071 | -1.288237 |
| H | -1.605971 | 0.43238 | -3.089812 |
| H | -3.214187 | 1.084065 | -2.754387 |
| H | -1.778269 | 1.921117 | -2.141774 |
| H | -3.913714 | 2.400722 | -0.825215 |
| H | -3.72302 | 2.605704 | 1.492504 |
| H | -5.117618 | 0.514822 | -1.653302 |
| H | -6.8456 | 2.458052 | -0.036299 |
| H | -7.431408 | 1.489478 | -1.40425 |
| H | -6.18012 | 2.711976 | -1.65231 |
| H | -5.07055 | -0.932864 | 0.382274 |
| H | -6.92694 | -0.909 | 1.980525 |
| H | -6.051536 | 0.638904 | 2.082224 |
| H | -7.61044 | 0.547441 | 1.252237 |
| H | -7.772896 | -0.725722 | -1.040044 |
| H | -7.118836 | -2.142198 | -0.214894 |
| H | -6.327901 | -1.529204 | -1.676643 |

**1c**

| C | -5.239729 | -1.7418 | -1.712893 |
| --- | --- | --- | --- |
| C | -5.675079 | -2.558358 | -0.504003 |
| C | -5.557925 | -1.852972 | 0.842224 |
| C | -4.208218 | -1.123112 | 0.969704 |
| C | -3.977661 | -0.088704 | -0.171324 |
| C | -3.953829 | -0.913811 | -1.494099 |
| C | -3.956132 | -0.508417 | 2.344695 |
| C | -2.473371 | -0.153023 | 2.447381 |
| C | -1.936553 | 0.568691 | 1.227354 |
| C | -2.63622 | 0.642357 | 0.062691 |
| C | -0.560033 | 1.223473 | 1.459046 |
| C | 0.071599 | 1.891685 | 0.180121 |
| C | -1.035313 | 2.544408 | -0.650101 |
| C | -2.142016 | 1.552101 | -0.998556 |
| C | 0.502161 | 0.155367 | 1.820157 |
| C | 0.913197 | -0.452607 | 0.471566 |
| C | 0.720185 | 0.683254 | -0.581647 |
| C | 1.067627 | 2.997235 | 0.578332 |
| C | -5.09028 | 0.990683 | -0.231338 |
| O | -6.088648 | -3.698161 | -0.602239 |
| O | -0.514219 | 3.111664 | -1.829936 |
| O | -0.801542 | 2.149855 | 2.52336 |
| O | -2.618284 | 1.623099 | -2.130757 |
| O | 2.278325 | -0.865818 | 0.423225 |
| C | 3.057979 | 0.17648 | -0.196226 |
| C | 2.120794 | 0.832593 | -1.240975 |
| C | 2.131001 | 0.207796 | -2.64432 |
| C | 4.367256 | -0.482346 | -0.67836 |
| O | 4.055263 | -1.69911 | -1.346056 |
| C | 5.386698 | -0.685776 | 0.481426 |
| C | 5.985947 | 0.662069 | 0.921885 |
| C | 6.467777 | -1.743312 | 0.120811 |
| C | 7.370621 | -1.333902 | -1.054051 |
| C | 7.310234 | -2.141316 | 1.343148 |
| H | -5.126723 | -2.422626 | -2.562083 |
| H | -6.072044 | -1.066688 | -1.961636 |
| H | -6.392773 | -1.145415 | 0.946787 |
| H | -5.672981 | -2.604215 | 1.630398 |
| H | -3.431971 | -1.894848 | 0.835381 |
| H | -3.801375 | -0.256673 | -2.349649 |
| H | -3.093229 | -1.595953 | -1.459103 |
| H | -4.57859 | 0.382542 | 2.49429 |
| H | -4.222612 | -1.215167 | 3.139912 |
| H | -2.267533 | 0.47413 | 3.321112 |
| H | -1.900291 | -1.079079 | 2.600597 |
| H | -1.515088 | 3.322439 | -0.0268 |
| H | 1.371663 | 0.664911 | 2.255802 |
| H | 0.165526 | -0.580431 | 2.552995 |
| H | 0.309552 | -1.337934 | 0.245832 |
| H | 0.021144 | 0.352643 | -1.354715 |
| H | 1.926823 | 2.62075 | 1.144647 |
| H | 1.446484 | 3.49612 | -0.315831 |
| H | 0.563107 | 3.758796 | 1.181698 |
| H | -6.07915 | 0.558799 | -0.4097 |
| H | -5.14312 | 1.564076 | 0.69982 |
| H | -4.889289 | 1.690637 | -1.046352 |
| H | -1.186921 | 2.9182 | -2.514199 |
| H | 0.059285 | 2.429667 | 2.872009 |
| H | 3.300769 | 0.930819 | 0.565246 |
| H | 2.394331 | 1.886037 | -1.344692 |
| H | 1.425532 | 0.75422 | -3.280648 |
| H | 3.120467 | 0.262136 | -3.107704 |
| H | 1.838664 | -0.846037 | -2.628107 |
| H | 4.823696 | 0.170363 | -1.43374 |
| H | 3.342149 | -2.098664 | -0.815933 |
| H | 4.821768 | -1.098768 | 1.331362 |
| H | 6.694859 | 0.53193 | 1.745636 |
| H | 5.217467 | 1.359451 | 1.273247 |
| H | 6.519294 | 1.153587 | 0.099436 |
| H | 5.9139 | -2.632607 | -0.199299 |
| H | 7.976336 | -0.449752 | -0.819792 |
| H | 8.065112 | -2.14651 | -1.29837 |
| H | 6.788132 | -1.121214 | -1.956706 |
| H | 7.955562 | -2.99462 | 1.10248 |
| H | 6.676432 | -2.432355 | 2.190315 |
| H | 7.964162 | -1.327366 | 1.679397 |

**1d**

| C | -5.511564 | -1.182556 | -1.797701 |
| --- | --- | --- | --- |
| C | -6.141081 | -1.841772 | -0.578164 |
| C | -5.918183 | -1.122499 | 0.747357 |
| C | -4.451976 | -0.677117 | 0.896138 |
| C | -3.986605 | 0.246049 | -0.268892 |
| C | -4.092301 | -0.619611 | -1.561621 |
| C | -4.113814 | -0.080624 | 2.260785 |
| C | -2.59262 | -0.032304 | 2.395844 |
| C | -1.89397 | 0.548588 | 1.181603 |
| C | -2.533552 | 0.709006 | -0.010887 |
| C | -0.432885 | 0.964069 | 1.458437 |
| C | 0.362544 | 1.451272 | 0.189193 |
| C | -0.57107 | 2.234928 | -0.737015 |
| C | -1.835564 | 1.450323 | -1.088163 |
| C | 0.407061 | -0.241204 | 1.924503 |
| C | 0.752147 | -0.988197 | 0.627246 |
| C | 0.819359 | 0.108395 | -0.48312 |
| C | 1.517924 | 2.385403 | 0.594152 |
| C | -4.859236 | 1.521599 | -0.404332 |
| O | -6.780923 | -2.873453 | -0.653295 |
| O | 0.092257 | 2.621925 | -1.915319 |
| O | -0.448699 | 1.918734 | 2.528464 |
| O | -2.247785 | 1.552803 | -2.242725 |
| O | 2.014275 | -1.648735 | 0.648956 |
| C | 2.998636 | -0.747759 | 0.100259 |
| C | 2.260753 | -0.030263 | -1.053346 |
| C | 2.231239 | -0.813657 | -2.375588 |
| C | 4.23604 | -1.6042 | -0.192108 |
| O | 4.596423 | -2.300226 | 1.002058 |
| C | 5.466131 | -0.843481 | -0.735443 |
| C | 6.570638 | -1.859376 | -1.079755 |
| C | 5.97059 | 0.346574 | 0.136313 |
| C | 6.705464 | -0.067409 | 1.422346 |
| C | 6.84323 | 1.304805 | -0.694354 |
| H | -5.51243 | -1.908648 | -2.616362 |
| H | -6.183265 | -0.366727 | -2.103852 |
| H | -6.594473 | -0.257427 | 0.800802 |
| H | -6.204326 | -1.803749 | 1.555306 |
| H | -3.846236 | -1.594385 | 0.809794 |
| H | -3.787674 | -0.041702 | -2.433371 |
| H | -3.38702 | -1.457209 | -1.472931 |
| H | -4.546865 | 0.922236 | 2.367764 |
| H | -4.537437 | -0.69247 | 3.066312 |
| H | -2.278023 | 0.541488 | 3.274163 |
| H | -2.223678 | -1.054701 | 2.563088 |
| H | -0.92709 | 3.134581 | -0.192533 |
| H | 1.319928 | 0.150743 | 2.384639 |
| H | -0.086508 | -0.864536 | 2.67246 |
| H | 0.003553 | -1.757439 | 0.41005 |
| H | 0.115838 | -0.130044 | -1.286135 |
| H | 2.253477 | 1.89655 | 1.238055 |
| H | 2.025723 | 2.756777 | -0.298599 |
| H | 1.134937 | 3.249916 | 1.143427 |
| H | -5.907716 | 1.28905 | -0.610637 |
| H | -4.829998 | 2.126405 | 0.508208 |
| H | -4.495335 | 2.139 | -1.229818 |
| H | -0.581359 | 2.528673 | -2.61962 |
| H | -0.972968 | 2.684794 | 2.241624 |
| H | 3.264315 | -0.01553 | 0.875774 |
| H | 2.710842 | 0.948011 | -1.238793 |
| H | 1.613747 | -0.278195 | -3.105078 |
| H | 3.22925 | -0.93101 | -2.809707 |
| H | 1.803944 | -1.813669 | -2.238226 |
| H | 3.946771 | -2.341347 | -0.958934 |
| H | 3.766042 | -2.665878 | 1.350849 |
| H | 5.129477 | -0.397458 | -1.683447 |
| H | 6.878832 | -2.421998 | -0.194793 |
| H | 7.45264 | -1.368369 | -1.503596 |
| H | 6.207958 | -2.581858 | -1.82075 |
| H | 5.085734 | 0.924345 | 0.438814 |
| H | 6.957794 | 0.820764 | 2.01494 |
| H | 6.095135 | -0.737031 | 2.03171 |
| H | 7.645611 | -0.58389 | 1.194219 |
| H | 7.775999 | 0.827883 | -1.018104 |
| H | 7.118065 | 2.185849 | -0.10237 |
| H | 6.318391 | 1.65594 | -1.591577 |

**1e**

| C | -5.240762 | -1.740831 | -1.712955 |
| --- | --- | --- | --- |
| C | -5.676428 | -2.557355 | -0.50415 |
| C | -5.558657 | -1.852307 | 0.842203 |
| C | -4.208632 | -1.123007 | 0.969575 |
| C | -3.977845 | -0.088501 | -0.171322 |
| C | -3.954477 | -0.91344 | -1.494191 |
| C | -3.956045 | -0.508717 | 2.344655 |
| C | -2.473113 | -0.153984 | 2.44712 |
| C | -1.936414 | 0.568137 | 1.227275 |
| C | -2.63618 | 0.642165 | 0.062704 |
| C | -0.559913 | 1.222951 | 1.459084 |
| C | 0.071792 | 1.891243 | 0.18028 |
| C | -1.035119 | 2.544178 | -0.649854 |
| C | -2.141841 | 1.551967 | -0.998458 |
| C | 0.502257 | 0.154881 | 1.820258 |
| C | 0.913509 | -0.45299 | 0.471657 |
| C | 0.720339 | 0.682856 | -0.581557 |
| C | 1.067801 | 2.99673 | 0.578702 |
| C | -5.09009 | 0.99131 | -0.231071 |
| O | -6.090716 | -3.696879 | -0.602549 |
| O | -0.514067 | 3.111614 | -1.829616 |
| O | -0.801542 | 2.149394 | 2.523361 |
| O | -2.617925 | 1.622956 | -2.13073 |
| O | 2.278748 | -0.865895 | 0.423401 |
| C | 3.058226 | 0.1764 | -0.196295 |
| C | 2.120868 | 0.832268 | -1.241014 |
| C | 2.131036 | 0.207266 | -2.644278 |
| C | 4.367534 | -0.482326 | -0.678452 |
| O | 4.055678 | -1.699142 | -1.346134 |
| C | 5.386995 | -0.685709 | 0.48131 |
| C | 5.985971 | 0.66214 | 0.922094 |
| C | 6.468306 | -1.742945 | 0.120489 |
| C | 7.371101 | -1.333127 | -1.054266 |
| C | 7.310861 | -2.141 | 1.34275 |
| H | -5.128163 | -2.421593 | -2.562254 |
| H | -6.072801 | -1.065318 | -1.961544 |
| H | -6.393199 | -1.14444 | 0.947124 |
| H | -5.673842 | -2.60367 | 1.630244 |
| H | -3.432727 | -1.895032 | 0.834954 |
| H | -3.801817 | -0.256281 | -2.349684 |
| H | -3.094167 | -1.595955 | -1.459348 |
| H | -4.578073 | 0.382491 | 2.494545 |
| H | -4.222698 | -1.21552 | 3.139768 |
| H | -2.266761 | 0.472612 | 3.321127 |
| H | -1.900393 | -1.080389 | 2.599622 |
| H | -1.514801 | 3.322128 | -0.026402 |
| H | 1.371723 | 0.664392 | 2.256016 |
| H | 0.165536 | -0.580996 | 2.552972 |
| H | 0.310093 | -1.338455 | 0.245873 |
| H | 0.02123 | 0.352182 | -1.354538 |
| H | 1.926999 | 2.620144 | 1.144943 |
| H | 1.446648 | 3.495794 | -0.315369 |
| H | 0.563248 | 3.758088 | 1.182275 |
| H | -6.079147 | 0.559826 | -0.409361 |
| H | -5.142588 | 1.564607 | 0.700164 |
| H | -4.888934 | 1.691296 | -1.04602 |
| H | -1.186553 | 2.917839 | -2.514003 |
| H | 0.059211 | 2.428121 | 2.873073 |
| H | 3.301009 | 0.930873 | 0.565046 |
| H | 2.394228 | 1.885742 | -1.344908 |
| H | 1.425523 | 0.753566 | -3.280661 |
| H | 3.120483 | 0.261537 | -3.1077 |
| H | 1.838717 | -0.846573 | -2.627874 |
| H | 4.823931 | 0.170405 | -1.433841 |
| H | 3.342785 | -2.098905 | -0.815878 |
| H | 4.822128 | -1.099008 | 1.331141 |
| H | 5.217365 | 1.35932 | 1.273578 |
| H | 6.519284 | 1.153932 | 0.099787 |
| H | 6.694864 | 0.531892 | 1.745842 |
| H | 5.914636 | -2.632303 | -0.199803 |
| H | 7.976495 | -0.448786 | -0.819898 |
| H | 8.065894 | -2.145479 | -1.298577 |
| H | 6.788634 | -1.120573 | -1.956968 |
| H | 7.964827 | -1.32706 | 1.678951 |
| H | 7.956155 | -2.994298 | 1.101985 |
| H | 6.677144 | -2.43206 | 2.189974 |

**1f**

| C | -5.424089 | -1.016381 | -1.798558 |
| --- | --- | --- | --- |
| C | -5.901522 | -1.99087 | -0.730783 |
| C | -5.647457 | -1.563567 | 0.71024 |
| C | -4.218373 | -1.017216 | 0.882757 |
| C | -3.91716 | 0.179902 | -0.06654 |
| C | -4.043219 | -0.384355 | -1.514576 |
| C | -3.840175 | -0.698144 | 2.327455 |
| C | -2.323282 | -0.527199 | 2.400217 |
| C | -1.757914 | 0.347129 | 1.298688 |
| C | -2.491849 | 0.70889 | 0.211562 |
| C | -0.306823 | 0.798611 | 1.558871 |
| C | 0.344261 | 1.621715 | 0.38432 |
| C | -0.71344 | 2.530342 | -0.245196 |
| C | -1.94026 | 1.742083 | -0.697556 |
| C | 0.6396 | -0.423855 | 1.655885 |
| C | 0.921855 | -0.81136 | 0.197249 |
| C | 0.8161 | 0.513566 | -0.620699 |
| C | 1.476693 | 2.52244 | 0.91257 |
| C | -4.899008 | 1.363803 | 0.136305 |
| O | -6.449289 | -3.041651 | -1.005833 |
| O | -0.179634 | 3.248299 | -1.333682 |
| O | -0.395894 | 1.532144 | 2.784946 |
| O | -2.451078 | 2.071152 | -1.767377 |
| O | 2.228187 | -1.348262 | -0.009609 |
| C | 3.096514 | -0.294979 | -0.471004 |
| C | 2.195057 | 0.630546 | -1.328352 |
| C | 2.074711 | 0.25236 | -2.812651 |
| C | 4.312983 | -0.955782 | -1.154106 |
| O | 3.883179 | -2.047051 | -1.960773 |
| C | 5.453565 | -1.404953 | -0.194307 |
| C | 4.99106 | -2.525233 | 0.752346 |
| C | 6.144862 | -0.234709 | 0.561445 |
| C | 7.382546 | -0.717597 | 1.339596 |
| C | 6.555541 | 0.925179 | -0.36484 |
| H | -5.425385 | -1.537083 | -2.761001 |
| H | -6.182224 | -0.222464 | -1.871458 |
| H | -6.389987 | -0.803432 | 0.991944 |
| H | -5.815611 | -2.431425 | 1.356287 |
| H | -3.542785 | -1.828739 | 0.564639 |
| H | -3.85114 | 0.399596 | -2.246413 |
| H | -3.26649 | -1.148584 | -1.655281 |
| H | -4.348836 | 0.21047 | 2.672889 |
| H | -4.153301 | -1.507764 | 2.997789 |
| H | -2.010238 | -0.0982 | 3.35784 |
| H | -1.85515 | -1.521049 | 2.348915 |
| H | -1.074569 | 3.225441 | 0.53612 |
| H | 1.578995 | -0.097959 | 2.12141 |
| H | 0.251481 | -1.243233 | 2.263962 |
| H | 0.212906 | -1.572151 | -0.145594 |
| H | 0.051407 | 0.407633 | -1.39496 |
| H | 2.307335 | 1.958918 | 1.351912 |
| H | 1.876281 | 3.132753 | 0.100298 |
| H | 1.087689 | 3.210673 | 1.669947 |
| H | -5.937991 | 1.079688 | -0.053477 |
| H | -4.845301 | 1.758591 | 1.156047 |
| H | -4.653068 | 2.176327 | -0.552102 |
| H | -0.897635 | 3.256738 | -1.998957 |
| H | 0.50511 | 1.650822 | 3.124062 |
| H | 3.449563 | 0.277807 | 0.398937 |
| H | 2.57983 | 1.652642 | -1.27325 |
| H | 1.401068 | 0.961095 | -3.307434 |
| H | 3.040971 | 0.288953 | -3.324203 |
| H | 1.674888 | -0.757012 | -2.947502 |
| H | 4.735458 | -0.224117 | -1.850346 |
| H | 3.133971 | -2.440063 | -1.479578 |
| H | 6.207732 | -1.83773 | -0.869536 |
| H | 4.474094 | -3.307421 | 0.189151 |
| H | 4.300354 | -2.152767 | 1.51752 |
| H | 5.838049 | -2.997528 | 1.258406 |
| H | 5.433136 | 0.162763 | 1.302226 |
| H | 8.117026 | -1.172063 | 0.661736 |
| H | 7.873345 | 0.124089 | 1.842347 |
| H | 7.13253 | -1.456729 | 2.106235 |
| H | 7.197173 | 0.568583 | -1.181246 |
| H | 7.122786 | 1.678785 | 0.193767 |
| H | 5.698104 | 1.435903 | -0.815444 |

## 2.2 ECD calculation of 2.

Conformation search of **2** at MMFF94s force field gave six conformers **2a**-**2f**. These conformers were optimized at B3LYP/6-31G(d) level, and then calculated the ECD at B3LYP/6-31G(d,p) level.

| Conformers | Gibbs free energies | Population (%) |
| --- | --- | --- |
| **2a** | －1115.421803 | 26.9 |
| **2b** | －1115.421853 | 28.4 |
| **2c** | －1115.421897 | 29.8 |
| **2d** | －1115.420209 | 5.0 |
| **2e** | －1115.420207 | 5.0 |
| **2f** | －1115.420203 | 4.9 |

Standard orientation of optimized **2** at B3LYP/6-31G(d) level.

**2a**

| C | 4.92085 | 2.84104 | 0.31560 |
| --- | --- | --- | --- |
| C | 4.76048 | 1.32932 | 0.23465 |
| C | 4.61559 | 0.80542 | -1.19769 |
| O | 3.58969 | 1.00158 | 0.98941 |
| C | 4.41100 | -0.71464 | -1.18884 |
| C | 3.31276 | -1.12760 | -0.21517 |
| C | 3.35171 | -0.39485 | 1.13827 |
| O | 2.03000 | -0.77182 | -0.74103 |
| C | 1.91041 | -0.55634 | 1.62428 |
| C | 1.08885 | -0.79281 | 0.33538 |
| C | -0.06403 | 0.15596 | -0.01284 |
| C | -1.04636 | -0.72940 | -0.82473 |
| C | -0.48558 | -2.18102 | -0.62180 |
| C | -1.64311 | -3.07916 | -0.18387 |
| C | -2.82792 | -2.15130 | 0.11592 |
| O | -3.89145 | -2.52614 | 0.59680 |
| C | -2.46234 | -0.79087 | -0.30116 |
| C | -3.46933 | 3.79224 | -0.10564 |
| C | -4.03843 | 2.43781 | -0.48504 |
| C | -4.97626 | 1.77942 | 0.53320 |
| C | -4.73934 | 0.27860 | 0.29400 |
| C | -3.31924 | 0.25340 | -0.21518 |
| O | -2.93585 | 1.49113 | -0.61552 |
| O | 0.47591 | -2.07133 | 0.43669 |
| H | 5.82507 | 3.16254 | -0.21290 |
| H | 4.05686 | 3.33900 | -0.13813 |
| H | 4.99255 | 3.16306 | 1.35929 |
| H | 5.63565 | 0.84384 | 0.70634 |
| H | 5.50336 | 1.06757 | -1.78697 |
| H | 3.75113 | 1.29705 | -1.65957 |
| H | 4.16738 | -1.09097 | -2.18892 |
| H | 5.34168 | -1.21036 | -0.87892 |
| H | 3.35003 | -2.21367 | -0.03450 |
| H | 4.09740 | -0.83359 | 1.82038 |
| H | 1.80144 | -1.42673 | 2.27688 |
| H | 1.59060 | 0.33478 | 2.16945 |
| H | -0.53823 | 0.50522 | 0.90874 |
| H | 0.29583 | 1.02601 | -0.56511 |
| H | -1.02398 | -0.44615 | -1.88301 |
| H | 0.01707 | -2.54481 | -1.52449 |
| H | -1.94373 | -3.78025 | -0.97260 |
| H | -1.38444 | -3.67490 | 0.69697 |
| H | -4.27546 | 4.53084 | -0.03338 |
| H | -2.95918 | 3.73751 | 0.86192 |
| H | -2.75260 | 4.13882 | -0.85664 |
| H | -4.52269 | 2.49379 | -1.47026 |
| H | -6.01749 | 2.08283 | 0.39369 |
| H | -4.67381 | 2.05879 | 1.54980 |
| H | -4.86564 | -0.35808 | 1.17045 |
| H | -5.41357 | -0.12096 | -0.47550 |

**2b**

| C | -4.69908 | 2.87873 | -0.64333 |
| --- | --- | --- | --- |
| C | -4.64230 | 1.40597 | -0.26228 |
| C | -4.34058 | 1.16923 | 1.22101 |
| O | -3.61534 | 0.81519 | -1.06533 |
| C | -4.25050 | -0.33408 | 1.51194 |
| C | -3.32907 | -1.04177 | 0.52434 |
| C | -3.50571 | -0.59922 | -0.93931 |
| O | -1.96500 | -0.71223 | 0.80361 |
| C | -2.16420 | -1.00361 | -1.54927 |
| C | -1.18078 | -1.00518 | -0.35662 |
| C | 0.02427 | -0.06407 | -0.36964 |
| C | 0.99804 | -0.73854 | 0.62511 |
| C | 0.53998 | -2.24026 | 0.60150 |
| C | 1.69798 | -3.07220 | 0.03495 |
| C | 2.89577 | -2.12243 | -0.10837 |
| O | 4.01722 | -2.47836 | -0.45260 |
| C | 2.45459 | -0.76415 | 0.23645 |
| C | 3.33956 | 3.84350 | -0.00339 |
| C | 3.90555 | 2.51866 | 0.47277 |
| C | 4.96226 | 1.86333 | -0.42383 |
| C | 4.74626 | 0.36285 | -0.16344 |
| C | 3.28327 | 0.30569 | 0.20054 |
| O | 2.82333 | 1.54110 | 0.51716 |
| O | -0.60589 | -2.30226 | -0.26144 |
| H | -5.49465 | 3.39039 | -0.09057 |
| H | -3.74576 | 3.36714 | -0.41316 |
| H | -4.89070 | 2.98981 | -1.71527 |
| H | -5.60794 | 0.92708 | -0.51166 |
| H | -5.11761 | 1.63226 | 1.84223 |
| H | -3.38742 | 1.65482 | 1.46182 |
| H | -3.89960 | -0.52304 | 2.53285 |
| H | -5.24843 | -0.78698 | 1.42799 |
| H | -3.46756 | -2.13312 | 0.58726 |
| H | -4.37059 | -1.08888 | -1.41487 |
| H | -2.20685 | -2.01169 | -1.97077 |
| H | -1.87098 | -0.30482 | -2.33636 |
| H | 0.45589 | -0.04757 | -1.37610 |
| H | -0.25132 | 0.95436 | -0.09011 |
| H | 0.85250 | -0.30544 | 1.62019 |
| H | 0.24849 | -2.57672 | 1.60236 |
| H | 1.97797 | -3.92490 | 0.66274 |
| H | 1.43255 | -3.47135 | -0.95108 |
| H | 4.12701 | 4.60531 | -0.01485 |
| H | 2.93379 | 3.74641 | -1.01597 |
| H | 2.53943 | 4.18695 | 0.65969 |
| H | 4.28364 | 2.61647 | 1.50029 |
| H | 5.97419 | 2.20419 | -0.18828 |
| H | 4.75779 | 2.10449 | -1.47406 |
| H | 4.97992 | -0.29400 | -1.00234 |
| H | 5.34998 | 0.00662 | 0.68204 |

**2c**

| C | 4.91589 | 2.84239 | 0.32742 |
| --- | --- | --- | --- |
| C | 4.75859 | 1.33107 | 0.23393 |
| C | 4.60561 | 0.81970 | -1.20208 |
| O | 3.59354 | 0.99372 | 0.99329 |
| C | 4.40470 | -0.70088 | -1.20558 |
| C | 3.31380 | -1.12528 | -0.22856 |
| C | 3.35978 | -0.40458 | 1.13110 |
| O | 2.02679 | -0.76806 | -0.74297 |
| C | 1.92206 | -0.57381 | 1.62493 |
| C | 1.09268 | -0.79997 | 0.33926 |
| C | -0.06363 | 0.14983 | 0.00694 |
| C | -1.04423 | -0.72786 | -0.81505 |
| C | -0.48684 | -2.18211 | -0.61970 |
| C | -1.64508 | -3.07851 | -0.17909 |
| C | -2.83122 | -2.15021 | 0.11424 |
| O | -3.89765 | -2.52509 | 0.58863 |
| C | -2.46272 | -0.78941 | -0.29892 |
| C | -3.46679 | 3.79416 | -0.09967 |
| C | -4.03501 | 2.44094 | -0.48469 |
| C | -4.97887 | 1.78143 | 0.52726 |
| C | -4.74192 | 0.28086 | 0.28648 |
| C | -3.31920 | 0.25541 | -0.21526 |
| O | -2.93258 | 1.49357 | -0.61104 |
| O | 0.48173 | -2.08004 | 0.43317 |
| H | 5.81587 | 3.17083 | -0.20404 |
| H | 4.04777 | 3.34228 | -0.11620 |
| H | 4.99362 | 3.15522 | 1.37346 |
| H | 5.63797 | 0.84351 | 0.69555 |
| H | 5.48888 | 1.08927 | -1.79475 |
| H | 3.73698 | 1.31334 | -1.65388 |
| H | 4.15548 | -1.06883 | -2.20739 |
| H | 5.33857 | -1.19709 | -0.90619 |
| H | 3.35492 | -2.21283 | -0.05788 |
| H | 4.11090 | -0.84764 | 1.80441 |
| H | 1.81920 | -1.45056 | 2.26997 |
| H | 1.60383 | 0.31143 | 2.18051 |
| H | -0.53711 | 0.48361 | 0.93471 |
| H | 0.29150 | 1.02914 | -0.53356 |
| H | -1.01569 | -0.43735 | -1.87118 |
| H | 0.00898 | -2.54430 | -1.52678 |
| H | -1.94393 | -3.78520 | -0.96338 |
| H | -1.38718 | -3.66823 | 0.70609 |
| H | -2.74583 | 4.14158 | -0.84621 |
| H | -4.27268 | 4.53329 | -0.03024 |
| H | -2.96181 | 3.73711 | 0.87047 |
| H | -4.51395 | 2.49923 | -1.47238 |
| H | -6.01908 | 2.08597 | 0.38271 |
| H | -4.68166 | 2.05863 | 1.54599 |
| H | -4.87336 | -0.35739 | 1.16105 |
| H | -5.41243 | -0.11671 | -0.48729 |

**2d**

| C | 4.98857 | 2.83627 | 0.17202 |
| --- | --- | --- | --- |
| C | 4.80321 | 1.32531 | 0.18572 |
| C | 4.68770 | 0.71052 | -1.21280 |
| O | 3.60685 | 1.06701 | 0.92722 |
| C | 4.45624 | -0.80217 | -1.11007 |
| C | 3.32531 | -1.13249 | -0.14232 |
| C | 3.34045 | -0.31261 | 1.16072 |
| O | 2.06357 | -0.79185 | -0.72604 |
| C | 1.88395 | -0.41929 | 1.61558 |
| C | 1.09341 | -0.72978 | 0.32267 |
| C | -0.03594 | 0.21068 | -0.12009 |
| C | -1.04083 | -0.71932 | -0.85120 |
| C | -0.47441 | -2.15634 | -0.58289 |
| C | -1.63288 | -3.04136 | -0.12567 |
| C | -2.78957 | -2.09273 | 0.21487 |
| O | -3.82329 | -2.43561 | 0.77777 |
| C | -2.43853 | -0.75393 | -0.27642 |
| C | -3.63295 | 3.40660 | 0.60242 |
| C | -4.01223 | 2.47434 | -0.54248 |
| C | -5.26969 | 1.62356 | -0.29611 |
| C | -4.70376 | 0.33221 | 0.32298 |
| C | -3.30252 | 0.28716 | -0.23153 |
| O | -2.94885 | 1.49441 | -0.73748 |
| O | 0.46176 | -1.99212 | 0.49071 |
| H | 5.91210 | 3.10749 | -0.35126 |
| H | 4.14601 | 3.31723 | -0.33698 |
| H | 5.03844 | 3.22515 | 1.19398 |
| H | 5.65645 | 0.85782 | 0.71253 |
| H | 5.59550 | 0.91917 | -1.79285 |
| H | 3.84480 | 1.18425 | -1.72993 |
| H | 4.23278 | -1.23984 | -2.08968 |
| H | 5.36946 | -1.29097 | -0.74257 |
| H | 3.33842 | -2.20467 | 0.11016 |
| H | 4.05966 | -0.71693 | 1.89083 |
| H | 1.74255 | -1.24191 | 2.32173 |
| H | 1.56487 | 0.51128 | 2.09069 |
| H | -0.50319 | 0.66235 | 0.75925 |
| H | 0.35158 | 1.01242 | -0.75154 |
| H | -1.05647 | -0.49602 | -1.92401 |
| H | 0.04994 | -2.55347 | -1.45889 |
| H | -1.96617 | -3.71845 | -0.92294 |
| H | -1.36433 | -3.66012 | 0.73561 |
| H | -4.36901 | 4.21366 | 0.69190 |
| H | -3.59567 | 2.86812 | 1.55618 |
| H | -2.65053 | 3.85390 | 0.42240 |
| H | -4.06587 | 3.03600 | -1.47972 |
| H | -5.76647 | 1.40253 | -1.24680 |
| H | -5.99102 | 2.13028 | 0.35097 |
| H | -4.66642 | 0.38072 | 1.41868 |
| H | -5.26096 | -0.57441 | 0.08232 |

**2e**

| C | 4.98844 | 2.83629 | 0.17220 |
| --- | --- | --- | --- |
| C | 4.80319 | 1.32533 | 0.18550 |
| C | 4.68726 | 0.71095 | -1.21317 |
| O | 3.60709 | 1.06672 | 0.92732 |
| C | 4.45592 | -0.80179 | -1.11079 |
| C | 3.32530 | -1.13246 | -0.14281 |
| C | 3.34084 | -0.31299 | 1.16049 |
| O | 2.06336 | -0.79167 | -0.72601 |
| C | 1.88452 | -0.41987 | 1.61581 |
| C | 1.09354 | -0.72995 | 0.32305 |
| C | -0.03596 | 0.21058 | -0.11903 |
| C | -1.04066 | -0.71917 | -0.85072 |
| C | -0.47437 | -2.15631 | -0.58262 |
| C | -1.63293 | -3.04128 | -0.12550 |
| C | -2.78975 | -2.09268 | 0.21465 |
| O | -3.82366 | -2.43559 | 0.77717 |
| C | -2.43856 | -0.75384 | -0.27644 |
| C | -3.63291 | 3.40630 | 0.60307 |
| C | -4.01205 | 2.47454 | -0.54228 |
| C | -5.26963 | 1.62379 | -0.29651 |
| C | -4.70398 | 0.33221 | 0.32236 |
| C | -3.30256 | 0.28724 | -0.23171 |
| O | -2.94874 | 1.49455 | -0.73743 |
| O | 0.46193 | -1.99235 | 0.49089 |
| H | 5.91178 | 3.10773 | -0.35130 |
| H | 4.14569 | 3.31736 | -0.33638 |
| H | 5.03861 | 3.22488 | 1.19426 |
| H | 5.65663 | 0.85773 | 0.71189 |
| H | 5.59486 | 0.91980 | -1.79345 |
| H | 3.84416 | 1.18477 | -1.72988 |
| H | 4.23222 | -1.23920 | -2.09047 |
| H | 5.36927 | -1.29063 | -0.74367 |
| H | 3.33852 | -2.20472 | 0.10932 |
| H | 4.06033 | -0.71749 | 1.89024 |
| H | 1.74337 | -1.24276 | 2.32170 |
| H | 1.56556 | 0.51052 | 2.09135 |
| H | -0.50328 | 0.66148 | 0.76067 |
| H | 0.35132 | 1.01287 | -0.74992 |
| H | -1.05591 | -0.49558 | -1.92348 |
| H | 0.04985 | -2.55335 | -1.45873 |
| H | -1.96601 | -3.71856 | -0.92269 |
| H | -1.36452 | -3.65987 | 0.73596 |
| H | -2.65039 | 3.85353 | 0.42345 |
| H | -4.36888 | 4.21342 | 0.69270 |
| H | -3.59592 | 2.86744 | 1.55663 |
| H | -4.06541 | 3.03657 | -1.47931 |
| H | -5.76616 | 1.40307 | -1.24740 |
| H | -5.99112 | 2.13039 | 0.35050 |
| H | -4.66704 | 0.38029 | 1.41809 |
| H | -5.26117 | -0.57428 | 0.08115 |

**2f**

| C | -4.98856 | 2.83619 | -0.17175 |
| --- | --- | --- | --- |
| C | -4.80319 | 1.32525 | -0.18554 |
| C | -4.68757 | 0.71038 | 1.21294 |
| O | -3.60688 | 1.06700 | -0.92713 |
| C | -4.45611 | -0.80231 | 1.11008 |
| C | -3.32521 | -1.13255 | 0.14228 |
| C | -3.34046 | -0.31259 | -1.16074 |
| O | -2.06345 | -0.79191 | 0.72593 |
| C | -1.88401 | -0.41921 | -1.61571 |
| C | -1.09334 | -0.72973 | -0.32289 |
| C | 0.03599 | 0.21071 | 0.11990 |
| C | 1.04076 | -0.71933 | 0.85115 |
| C | 0.47439 | -2.15632 | 0.58268 |
| C | 1.63293 | -3.04132 | 0.12560 |
| C | 2.78966 | -2.09266 | -0.21475 |
| O | 3.82344 | -2.43550 | -0.77755 |
| C | 2.43856 | -0.75390 | 0.27659 |
| C | 3.63245 | 3.40622 | -0.60294 |
| C | 4.01203 | 2.47451 | 0.54230 |
| C | 5.26958 | 1.62384 | 0.29613 |
| C | 4.70383 | 0.33223 | -0.32260 |
| C | 3.30254 | 0.28720 | 0.23181 |
| O | 2.94884 | 1.49443 | 0.73779 |
| O | -0.46165 | -1.99202 | -0.49104 |
| H | -5.91208 | 3.10736 | 0.35156 |
| H | -4.14600 | 3.31714 | 0.33726 |
| H | -5.03845 | 3.22514 | -1.19369 |
| H | -5.65646 | 0.85778 | -0.71233 |
| H | -5.59532 | 0.91896 | 1.79310 |
| H | -3.84462 | 1.18406 | 1.73002 |
| H | -4.23266 | -1.24005 | 2.08967 |
| H | -5.36934 | -1.29106 | 0.74254 |
| H | -3.33832 | -2.20471 | -0.11027 |
| H | -4.05973 | -0.71688 | -1.89081 |
| H | -1.74264 | -1.24181 | -2.32189 |
| H | -1.56500 | 0.51139 | -2.09083 |
| H | 0.50336 | 0.66223 | -0.75946 |
| H | -0.35150 | 1.01256 | 0.75122 |
| H | 1.05622 | -0.49609 | 1.92398 |
| H | -0.05010 | -2.55348 | 1.45857 |
| H | 1.96612 | -3.71841 | 0.92292 |
| H | 1.36451 | -3.66010 | -0.73571 |
| H | 4.36839 | 4.21333 | -0.69291 |
| H | 3.59507 | 2.86732 | -1.55646 |
| H | 2.65000 | 3.85348 | -0.42295 |
| H | 4.06562 | 3.03655 | 1.47931 |
| H | 5.76640 | 1.40311 | 1.24687 |
| H | 5.99085 | 2.13049 | -0.35108 |
| H | 4.66665 | 0.38026 | -1.41833 |
| H | 5.26112 | -0.57422 | -0.08148 |
